# Assessing the risk of an emerging zoonosis of worldwide concern: anisakiasis

**DOI:** 10.1038/srep43699

**Published:** 2017-03-13

**Authors:** Miguel Bao, Graham J. Pierce, Santiago Pascual, Miguel González-Muñoz, Simonetta Mattiucci, Ivona Mladineo, Paolo Cipriani, Ivana Bušelić, Norval J. C. Strachan

**Affiliations:** 1 Oceanlab, University of Aberdeen, Main Street, Newburgh, Aberdeenshire, AB41 6AA, United Kingdom; 2 College of Physical Sciences, School of Natural and Computing Sciences, University of Aberdeen, St. Machar Drive, Cruickshank Bd., Aberdeen AB24 3UU, United Kingdom; 3 Centre for Environmental and Marine Studies (CESAM) & Departamento de Biologia, Universidade de Aveiro, Campus Universitário de Santiago, 3810-193 Aveiro, Portugal; 4 ECOBIOMAR, Instituto de Investigaciones Marinas (CSIC), Eduardo Cabello 6, E-36208 Vigo, Spain; 5 University Hospital La Paz-Institute for Biomedical Research (IdiPaz), Paseo de la Castellana 261, 28046 Madrid, Spain; 6 Department of Public Health and Infectious Diseases, Section of Parasitology, Sapienza University of Rome, P. le Aldo Moro, 5 00185 Rome, Italy; 7 Institute of Oceanography and Fisheries, Laboratory of Aquaculture, Šetalište Ivana Meštrovića 63, 21000 Split, Croatia; 8 Department of Ecology and Biology (DEB), Tuscia University, Viale dell’Università, snc 01100 Viterbo, Italy; 9 College of Life Sciences and Medicine, School of Biological Sciences, University of Aberdeen. St. Machar Drive, Cruickshank Bd., Aberdeen, AB24 3UU, United Kingdom

## Abstract

Anisakiasis is an emerging zoonosis caused by the fish parasitic nematode *Anisakis*. Spain appears to have the highest reported incidence in Europe and marinated anchovies are recognised as the main food vehicle. Using data on fishery landings, fish infection rates and consumption habits of the Spanish population from questionnaires, we developed a quantitative risk assessment (QRA) model for the anchovy value chain. Spaniards were estimated to consume on average 0.66 *Anisakis* per untreated (non-frozen) raw or marinated anchovy meal. A dose-response relationship was generated and the probability of anisakiasis was calculated to be 9.56 × 10^−5^ per meal, and the number of annual anisakiasis cases requiring medical attention was predicted between 7,700 and 8,320. Monte Carlo simulations estimated *post-mortem* migration of *Anisakis* from viscera to flesh increases the disease burden by >1000% whilst an education campaign to freeze anchovy before consumption may reduce cases by 80%. However, most of the questionnaire respondents who ate untreated meals knew how to prevent *Anisakis* infection. The QRA suggests that previously reported figures of 500 anisakiasis per year in Europe is a considerable underestimate. The QRA tool can be used by policy makers and informs industry, health professionals and consumers about this underdiagnosed zoonosis.

Most emerging infectious diseases are of zoonotic origin and consequently involve spill-over from animal to human populations^1^,^2^. A number of factors can contribute to the emergence of an infectious disease, such as ecological changes (including those due to economic development and agricultural land use or changes in marine activity), human demographics and behaviour, international travel and commerce, technology and industry, microbial adaptation/change, and breakdown in public health measures^3^.

*Anisakis* spp. are nematode parasites found in a wide range of marine organisms. Their life cycle ecology involves cetaceans as final hosts, zooplankton as intermediate hosts and fish and cephalopods as intermediate or paratenic hosts (i.e. transport host in which survival but no larval development occurs, even though this phase may be crucial for successful transfer of the parasite to the definitive host and completion of its life cycle^4^,^5^). Within the *Anisakis* spp. life cycle, humans may become accidental hosts in which the parasite can survive for a short period of time but cannot reproduce^6^,^7^. Humans become infected by eating raw or undercooked fish that contain viable *Anisakis* spp. third stage larvae^6^. The ingestion of live *Anisakis* spp. larvae may cause human disease (i.e. anisakiasis). The severity of anisakiasis varies from mild to severe and can have gastric, intestinal, ectopic and allergic forms^4^,^6^,^7^,^8^. The disease is thought to be frequently misdiagnosed and underdiagnosed since symptoms of anisakiasis are usually not specific^4^,^9^,^10^,^11^, with rare outbreaks^12^ and person to person transmission non-feasible.

The genus *Anisakis* comprises nine species of which two (*A. simplex* s.s. and *A. pegreffii*) have been confirmed as zoonotic pathogens^5^. Anisakiasis is an emerging human health problem and is also of economic concern because of the potential negative effects on consumer confidence and the marketability problems associated with infested fishery products^4^,^5^,^8^,^13^,^14^. Recent increases in medical case reports of anisakiasis have been observed in a number of countries around the world, and may be due to improved public health diagnoses (i.e. improved techniques and expertise), and/or behaviour change through increasing global demand for seafood and a growing preference for raw or lightly cooked food, especially in many Western countries^4^,^13^. The European Food Safety Authority (EFSA) Panel on Biological Hazards (BIOHAZ) reported approximately 20,000 anisakiasis cases worldwide prior to 2010, with > 90% from Japan^4^. In Europe, Spain is considered to have the highest incidence of anisakiasis, predominantly through consumption of the traditional marinated dish “anchovies in vinegar”^4^. However the actual anisakiasis burden in the human population is unknown because of the scarcity of epidemiological data^4^. There is a need to determine and understand the burden of disease associated with this zoonotic nematode in the human population and to identify measures to reduce its incidence.

European anchovy (*Engraulis encrasicolus*), which is the anchovy species usually consumed in Spain, is a small pelagic (i.e. living in the water column) marine fish which tends to aggregate in large shoals, especially near the coast^15^. Its distribution in the Eastern North and Central Atlantic Ocean extends from the North Sea to South Africa and it is also found throughout the Mediterranean and in the Black and Azov Seas^15^. This species is of high economic interest in Spain with total capture production of 36,148 t reported in 2013 (Food and Agriculture Organization of the United Nations (FAO))^16^. Imports of fresh or refrigerated anchovy into Spain derive mainly from Italy, France and Morocco (totalling 8,757 t in 2013) (Ministerio de Agricultura, Alimentación y Medio Ambiente (MAGRAMA, *pers. comm.*)). There are also exports from Spain, mainly to Italy and Morocco (2,949 t in 2013) (MAGRAMA, *pers. comm.*). Spanish production, imports and exports can vary considerably year on year^17^. *Anisakis simplex* s.l. (probably belonging to *A. simplex* s.s. and *A. pegreffii*, since mixed infection of both *Anisakis* species are commonly found in fish species inhabiting the Iberian Atlantic coast^5^) and *A. pegreffii* have been reported in European anchovy from the Gulf of Cádiz and Strait of Gibraltar (Iberian Atlantic coast)^18^, and from Mediterranean Sea^19^,^20^, respectively, and their prevalence and intensity of infection in this fish may vary depending on the fishing area and season^18^,^19^,^20^.

Quantitative risk assessment (QRA) is a science-based methodology that estimates the probability and severity of an adverse event^21^, e.g. health risk to individuals or populations due to exposure of zoonotic parasites through ingestion of contaminated fish meals. Used alongside Monte Carlo simulation methods, QRA estimates the human health risk simulating the uncertainty (lack of knowledge) and variability of the associated model parameters^22^. The process involves four main stages^23^,^24^:

(1) Hazard identification: identifies the pathogen (e.g. *Anisakis* spp.) of concern, determines whether it is actually a hazard, and identifies the vehicle of transmission (e.g. raw and marinated anchovies).

(2) Exposure assessment: determines the number of *Anisakis* spp. ingested per meal (i.e. the dose).

(3) Hazard characterization: gives a quantitative or qualitative assessment of the adverse effects of the pathogen on humans; more specifically a dose-response model can be implemented, which mathematically models the response (i.e. the impact and its variability) following exposure to different doses.

(4) Risk characterization: gives a probability of occurrence of the disease (e.g. anisakiasis) and estimates the disease burden in a given population.

Quantitative microbiological risk assessment has been performed to estimate health risk in humans from exposure to microbes, e.g. *Escherichia coli* O157 from eating beef burgers^21^ and from recreational use of animal pasture^25^, *Cryptosporidium parvum* in drinking water^26^ and *Listeria monocytogenes* in smoked salmon and trout^27^. A number of studies have been carried out to determine the levels of *Anisakis* spp. infection in fish species and then assess the food safety implications of these findings^18^,^19^,^20^,^28^,^29^. However, to date no QRA for *Anisakis* spp. (or any other marine zoonotic parasite) in a fish meal is available.

The present study aims to integrate data obtained from social science methods (questionnaires and economic surveys) and from the natural sciences (fish parasite sampling surveys and infection rates in humans) in a process-based QRA model^21^. This is then used to determine the probability of disease (i.e. anisakiasis) caused by consumption of untreated (i.e. not previously frozen) raw and marinated anchovy meals prepared at home in Spain (hereinafter: untreated anchovy meals). The burden of disease for the whole Spanish population will then be estimated, and the effects of hypothetical scenarios, i.e. factors that can increase risk (*post-mortem* migration of *Anisakis* spp. from fish viscera to muscle) and interventions to decrease risk (public health education campaign).

Since this is a QRA study it does not follow the typical steps of a scientific paper^21^,^25^,^27^, as it gives methods, results and discussion through the steps of the QRA section. This is done for ease of interpretation of the QRA model.

## QRA Section

### Materials and methods

#### Value Chain of Anchovies

**Figure 1:**
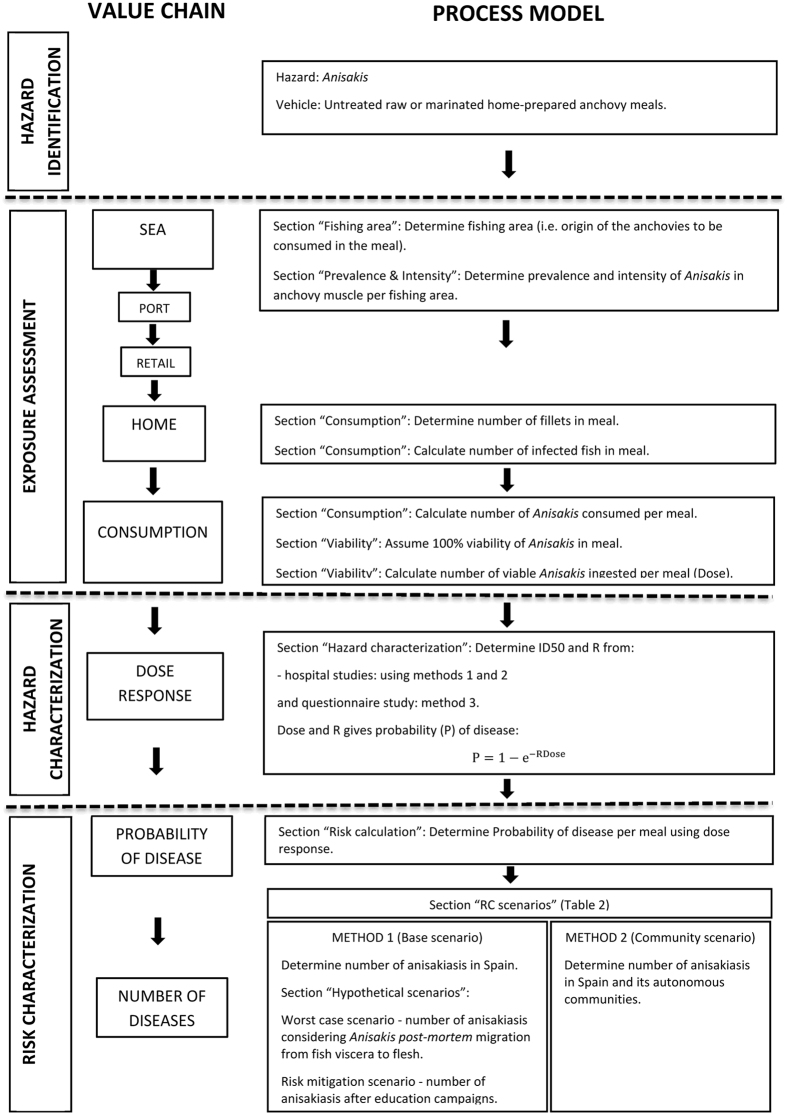
Value chain and process model outline for untreated raw and marinated home-prepared anchovy.

The value chain for anchovies, from sea to consumption, was characterised and from this a process model was generated which incorporated the four steps of a risk assessment, i.e. hazard identification, exposure assessment, hazard characterization and risk characterization (Fig. 1). The QRA model was implemented in Microsoft Excel™ using @RISK software (Palisade, UK) and parameterized as detailed in Tables 1 and 2.

#### Data Collection

##### *Anisakis* spp. Infection Descriptors in European Anchovy and Anchovy Biometrics

Parasite infection parameters (i.e. prevalence and number of *Anisakis* spp. in anchovy muscle (intensity of infection)) and other data on the fish (i.e. fishing area and total weight of fish caught) were obtained from our own sampling and from the literature (Rello *et al*.^18^) (see Table 3 for further details):

European anchovies (n = 3,799) caught in FAO areas 37 (Mediterranean and Black Sea) and 27 (Atlantic, Northeast) (areas 1 and 2 respectively in the model) were immediately frozen after capture and later inspected for *Anisakis* spp. in muscle. The methodology of parasite inspection followed Karl and Leinemann^30^. Briefly, after thawing, anchovy were filleted and the fillets were pressed and then frozen prior to further examination. Visual inspection was then performed on thawed anchovy fillets using a 366 nm UV-light source, which caused dead *Anisakis* spp. larvae present in the fillets to fluoresce.

Data on *Anisakis* spp. infection of anchovy from FAO area 34 (Atlantic, Eastern Central (area 3 in the model)) were obtained from Rello *et al*.^18^ (Table 3). The number of *Anisakis* spp. larvae found in the muscle of each fish was estimated from Rello *et al*.^18^ as follows: the prevalence of infection of the 396 anchovies inspected was 13.13%, hence 52 anchovies were infected. The number of larvae in the muscle of fish was calculated to be 67 by multiplying the number of anchovies with muscle infection (52) by the mean intensity (1.28). Since the intensity range was from 1 to 4 larvae (see Table 1 Rello *et al*.^18^), it was then possible to estimate the numbers and hence the distribution of *Anisakis* spp. in the flesh of each fish (Table 4). Of the 396 anchovies inspected, 344 anchovies had no *Anisakis* spp. larva in the muscle, 42 had 1 larva, 6 had 2, 3 had 3 and 1 had 4 (Table 4).

##### Anchovy Consumption Habits of the Spanish Population

Consumption data from questionnaires: Online questionnaires (see Supplementary materials) were made available to the Spanish population in order to gather information about anchovy consumption habits (questionnaire 1), and to estimate the number of fillets eaten by Spanish consumers in each anchovy in vinegar meal (questionnaire 2). Questionnaire 1 was advertised on social media, the website of the EU FP7 PARASITE project (GA no. 312068), websites of research institutions and regional government of the autonomous community of Galicia (NW Spain); in the local press - “Faro de Vigo” located at the NW of Spain, a fish industry journal - “Industrias pesqueras”, local and national radio programmes, by emails sent to research, food safety and consumption institutions, personal/professional contacts, and “word of mouth”. The second questionnaire was disseminated by emails sent to personal or professional contacts and by “word of mouth”.

The data collected were used in the risk model (see “Risk assessment” section) to determine the total numbers of anchovy meals and untreated anchovy meals consumed by survey respondents, and to then estimate the number of untreated anchovy meals consumed by the Spanish population. This was used in risk characterization methods 1 and 2 (details in “RC scenarios” section), as well as in the dose response calculations (details in Hazard characterization” section). Respondents with allergy to *Anisakis* spp. were also identified and this information was used in dose-response method 3 (details in “Dose-response method 3” section).

Consumption data from governmental estimates: The total mass of fresh anchovies consumed at home in 2013 by the Spanish population was available from governmental estimates^31^, and used to determine the total number of untreated anchovy meals consumed in Spain and in each of its autonomous communities in risk characterization method 2 (Community scenario) (details in “RC scenarios” section), and in dose-response method 2 (details in “Dose-response method 2” at “Hospital studies” section).

##### Landing Statistics and Trade Data

Spanish global fishery production statistics and trade (i.e. imports and exports) by supplier countries of European anchovy were obtained from FAO^16^ and MAGRAMA (*pers. comm.*), respectively. These data were used to determine the fishing area (i.e. origin) of the anchovies consumed in Spain, since *Anisakis* spp. infection rates in anchovy vary with area (details in “Fishing area” section).

#### QRA Methodology

A process-based risk assessment model was implemented in Microsoft Excel™ using @RISK software (Palisade, UK). This incorporated variability in input data through the use of appropriate probability distributions in simulations (n = 100,000) using the Monte Carlo technique^22^. The QRA model was performed for the risk assessment process detailed in Fig. 1 and parameterised according to Tables 1 and 2. Each Monte Carlo iteration determined the probability of disease from consuming one untreated anchovy meal.

#### Risk Characterization and Hypothetical Scenarios

The burden of disease (i.e. total number of anisakiasis cases and anisakiasis incidence per year) was estimated by two risk characterization methods:

Method 1 or Base scenario: the number of untreated anchovy meals consumed by the Spanish population was calculated using questionnaire 1 data.

Method 2 or Community scenario: the number of untreated anchovy meals consumed by Spaniards was calculated using data on the mass of fresh whole anchovy home-consumed in Spain available from MAGRAMA^31^.

The QRA model was modified to determine the potential increase in risk (i.e. increase in number of anisakiasis cases per year) considering a worst case scenario of *post-mortem* migration of *Anisakis* spp. from fish viscera (where the majority of worms are found while the fish is alive) to muscle; such *post-mortem* migration of *Anisakis* spp. is well-documented in anchovy^19^,^20^. The QRA model was also modified to determine the efficacy of a risk mitigation scenario i.e. education campaign to freeze anchovies to reduce the number of diseases per year. The latter follows Spanish legislation which came into effect in 2006 (Royal Decree RD 1420/2006)^32^ implemented to improve the risk control of anisakiasis in Spain. Consumers’ attitudes were investigated from questionnaire responses to determine the likelihood they would respond to an education campaign. Both hypothetical scenarios were determined using risk characterization method 1 (Base scenario).

### Risk assessment

#### Hazard Identification

The first probable case of anisakiasis was reported in 1876 in Greenland^4^. In the Netherlands, during the 1960 s, Van Thiel described anisakiasis as “worm-herring disease”^33^. Currently, thousands of cases are diagnosed every year, particularly in Japan (approximately 2,000 to 3,000 cases^4^,^34^), Spain, the Netherlands, Germany^4^, Korea^35^ and Italy^36^ where eating raw or marinated fish is common. Recently, cases were reported in Croatia^37^, China^38^ and Taiwan^39^. *Anisakis simplex* s.s. and *A. pegreffii* are the zoonotic species involved. Anisakiasis is therefore a human zoonotic disease caused by certain species of nematodes belonging to genus *Anisakis*^6^. It is an increasing health problem worldwide^4^,^8^,^13^ and its severity may vary from mild to severe gastrointestinal and ectopic disorders as well as allergy^4^,^6^,^7^,^8^. The work in this paper will concentrate on the gastric, intestinal and gastroallergic anisakiasis forms of disease.

In Spain, European anchovy is traditionally consumed marinated as “anchovies in vinegar” and is the main fish specialty implicated as the cause of human anisakiasis^6^,^9^,^18^,^40^,^41^,^42^. The hazard associated with this source of infection is the zoonotic nematode *Anisakis* spp. that can survive in marinated and raw fish products for a sufficient period of time before being ingested^4^. Since Spanish legislation requires restaurants to freeze the fish presented as raw or almost raw, before serving^32^, following the guideline previously established in Regulation (EC) 853/2004^43^, the main risk of disease is from the consumption of untreated raw or marinated anchovy at home. Freezing or thorough cooking are considered as preventive methods to inactivate any possible *Anisakis* spp. present in fishery products^4^. Other anchovy species (e.g. *Engraulis anchoita* and *E. ringens*) can be consumed in Spain, but usually are imported as frozen, salted or canned^17^ and were therefore excluded from this study.

Hence, *Anisakis* spp. is considered as the hazard and untreated raw or marinated European anchovies as the main etiological vehicle involved in anisakiasis in Spain.

#### Exposure Assessment

In order to determine the risk of anisakiasis caused by consumption of untreated anchovy meals, the potential exposure to the parasite from each meal needs to be determined. This depends on: 1) the fishing area of anchovies consumed in Spain (see section “Fishing area” below); 2) the prevalence and intensity of *Anisakis* spp. in anchovy muscle for each fishing area (see section “Prevalence & Intensity” below); 3) the consumption of untreated anchovy meals by the Spanish population (see section “Consumption” below) and 4) viability of *Anisakis* spp. in untreated anchovy meals (see section “Viability” below).

##### Fishing Area - The Fishing Area of Anchovies Consumed in Spain

Anchovies from different fishing areas are expected to have different *Anisakis* spp. burdens and therefore, the risk for consumers will vary. Considered first are the anchovy imported into Spain and then anchovy caught by Spanish vessels.

Fishing Area of Anchovy Imported into Spain: Fresh anchovies are imported every year into Spain from supplier countries (Table S1) and exports also occur (Export, Table S2) (MAGRAMA, *pers. comm.*). The quantities and countries involved in anchovy transactions are known. To determine the mass, per fishing area, of anchovy imports (Assumed imports, Table S1), it was assumed that the imports per fishing area are in the same proportions as seen in landings of the supplier country (Production, Table S1)^16^.

Total exports from Spain are known, but the fishing ground of exported anchovies was unknown. It was assumed that exports comprised fish from the three different fishing areas considered in the present study in proportion to the amount caught (Table S2).

Only fresh imported and exported anchovies were considered, since other anchovy products (i.e. frozen, canned, salted or semi-preserved, etc.) were assumed not to contain viable *Anisakis* spp. The trade (i.e. imports and exports) data were provided for fresh anchovy, identified as *Engraulis* spp. by MAGRAMA (*pers. comm.*). However, it was assumed these data refer to European anchovy (*E. encrasicolus*) since it is the only anchovy species fished by supplier countries^16^.

Fishing Area of Anchovy Fished by Spanish Vessels: The Spanish production of European anchovy by fishing area was directly available from FAO^16^ (Table S2).

The total quantities of anchovies from each fishing area (Table S2) were the result of summing “Spanish Production” and “Imports” and then subtracting “Exports”. The proportion of estimated consumption for each fishing area was then calculated (Table S2). This was then used in the QRA model to determine the fishing area of each untreated anchovy meal by randomly sampling the RiskDiscrete distribution (the first argument of this @RISK distribution is the set of possible values (i.e. fishing areas), and the second is the set of corresponding probabilities (the probability of the fish originating from a particular area)) (Area, Table 1). Thus, the QRA model starts by selecting the fishing area of origin for each anchovy meal (1, 2 or 3).

##### Prevalence & Intensity - Prevalence and Intensity of Infection of *Anisakis* Spp. in Anchovy Muscle for Each Fishing Area

The prevalence of *Anisakis* spp. in the muscle of anchovy for each of the three fishing areas is given by the variables “Parea1”, “Parea2” and “Parea3” (Table 1). The corresponding numbers of *Anisakis* spp. in anchovy muscle in each fishing area (NparasitesArea1, 2 and 3, Table 1) are described by a discrete distribution using the data from Table 4.

The fishing area is selected by sampling the RiskDiscrete variable “Area” whose output “1”, “2” or “3” selects the corresponding prevalence “Pselected” and also the number of *Anisakis* spp. in muscle per infected anchovy (NparasitesArea1, 2 and 3).

The QRA model was performed for 2013, the most recent year for which production and trade data were available. Anchovy infection data (Tables 3 and 4) were assumed to be representative for 2013, even though samples were obtained from different years (Table 3).

##### Consumption - Consumption of Untreated Raw and Marinated Anchovy Meals by the Spanish Population

###### Estimating the fraction of untreated anchovy meals eaten:

A total of 729 completed questionnaires was obtained from questionnaire 1. Those respondents (n = 13) who did not respond to question 18 (i.e. Do you usually eat raw or undercooked fish?) were removed. The remaining respondents (n = 716) consumed a total of 5,767 anchovy meals per year (question 16). It was assumed that a respondent consumed untreated anchovy meals when s/he gave all of the following responses (Table S3): (1) answer “yes” to eating raw fish in question 18; (2) eating raw fish at their home (question 18 A); (3) utilising fresh fish for raw/undercooked consumption (question 18A2); (4) confirming raw anchovy as the consumed species (question 18B), and finally (5) indicating the specific kind of raw and/or marinated anchovy preparation that they consumed (question 18C1). These “at risk” respondents (n = 46) ate a total of 987 untreated home-prepared raw or marinated anchovy meals per year (i.e. anchovies in vinegar or lemon (472), marinated anchovies (280), sushi or sashimi (106), ceviche (80) and carpaccio (49)) (question 18C1). It was assumed that a respondent ate 1, 12 or 52 meals per year when s/he answered annually, monthly or weekly anchovy consumption in questionnaire 1 (questions 16 and 18C1). The probability of an untreated anchovy meal being consumed was 0.17 (=987/5,767). Finally, it was assumed that these meals were the main vehicle of anisakiasis in Spain (see section “Hospital studies” and “Anchovy main species causing anisakiasis” at the “General discussion” section for further details).

Questionnaire 1 was over-represented by respondents from Galicia (62%, n = 447), whose population is approximately 6% of Spain (Spanish Statistical Office - available at: http://www.ine.es/accessed 05^th^ Dec 2016). The questionnaire reported that the number of untreated anchovy meals consumed per Galician respondent per year (0.74 = 332/447) was a third of that from other communities in Spain (2.48 = 653/263). To correct for this bias the respondents were grouped according to geographical location (i.e. Galicia, Cantabrian Sea, Central Spain and Mediterranean Sea communities, Andalucía and Canary Islands) and their responses were weighted based on the population of each region, to provide representative results for Spain, (see Table S4). As a result, the number of untreated meals consumed per Spanish person per year was estimated to be 2.106 (Amealpy, Table S4) and the total number of untreated meals consumed by respondents was 1,508 (i.e. 716 × 2.106 = 1,508) (Table S4). Hence, the proportion of untreated meals consumed per person was 0.175 (Propunt, Table S4). These figures were used when calculating the dose-response for *Anisakis* spp. (see “Hazard characterization” section) and during Risk Characterization (see “Risk characterization” section).

Estimating a probability distribution for the number of fillets consumed in an anchovy meal: A separate model (i.e. “anchovy meal size” sub-model) was built to estimate the number of anchovy in vinegar fillets consumed per meal (NFillet, Table 1) using data from questionnaire 2. Detailed information of the sub-model is provided in the Supplementary material.

In the QRA model, the number of fish in a meal was determined knowing that there are two fillets per fish. The number of fish with at least one parasite present was then calculated using the RiskBinomial distribution (Nfishinf, see Table 1).

The exposure to parasites (i.e. number of *Anisakis* spp. consumed per untreated anchovy meal) (Nparasite, Table 1) was then determined by multiplying the number of parasites in muscle of an infected anchovy (NparasitesArea1, 2 and 3, Table 1) by the number of infected anchovies consumed per meal (Nfishinf, Table 1). This approximation was required for the model to be readily implemented in Excel using @RISK. However, *Anisakis* spp. exposure was also determined by sampling each infected anchovy consumed in the meal and using the Monte Carlo method to generate a distribution of the infected anchovies in each meal. This distribution was compared with the approximate method described above and was found to have virtually identical mean and variance (data not presented).

##### Viability - Viability of *Anisakis* Spp. in Untreated Anchovy Meals

It has been reported that *Anisakis* spp. can survive the typical conditions experienced in the traditional preparation of anchovies in vinegar^44^, even though it is unknown what proportion of parasites is viable in freshly caught anchovy. In the first instance, the QRA model assumed that all *Anisakis* spp. present in untreated anchovy meals had 100% viability (Propviable, Table 1). However, to investigate the effect of reduced viability, simulations were also carried out with viabilities of 50% and 10% in determination of the dose response.

The number of viable *Anisakis* spp. in the untreated anchovy meal (Dose, Table 1) was then calculated by multiplying “Nparasite” by “Propviable” (Table 1).

#### Hazard Characterization

No dose-response model has been developed previously for *Anisakis* spp. in humans. Typically, if it is assumed that the distribution of the pathogen (i.e. *Anisakis* spp.) within a fish is Poisson, that one organism is sufficient to have the potential to cause disease and that each organism has an equal and identical survival probability (R) (i.e. the probability of colonising and causing disease), then the form of the dose response is exponential^45^:


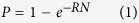


where P, is the probability of disease following consumption of a dose of N pathogens (e.g. *Anisakis* spp.). The ID50 (the dose of an infectious organism required to produce disease in 50 percent of subjects challenged) is given by:


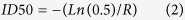


Here, data from four studies performed in Spanish hospitals, our own sampling, questionnaires and MAGRAMA^31^ were used to estimate R and the ID50 using two different methods. A third independent method for determining the dose-response using questionnaire 1 information (e.g. respondents with self-reported allergy to *Anisakis* spp.) was also performed. These data were then used in the QRA model to convert the number of parasites ingested in a meal to the probability of disease from consuming an untreated anchovy meal (Pdisunt, Table 1).

##### Hospital Studies

In 1997, 96 patients (incidence of 19.2 cases per 100,000 inhabitants/year, Table S9) were diagnosed with gastroallergic anisakiasis in “La Paz” hospital in Madrid (central part of Spain)^40^. Seventy-eight of these patients confirmed ingestion of raw anchovies in vinegar (n = 76) or raw anchovies (n = 2) prior to falling ill^40^. A retrospective study performed in “Virgen de la Salud” hospital in Toledo (central part of Spain) reported 25 cases (3.87 cases per 100,000 inhabitants/year, Table S9) of gastrointestinal anisakiasis over two years (December 1999 to January 2002)^46^. All these patients confirmed ingestion of raw anchovies prior to falling ill^46^. A third study, performed in “Antequera” hospital in Málaga (south of Spain) reported 52 patients (11.82 cases per 100,000 inhabitants/year, Table S9) with anisakiasis over a four-year period (summer 1999 to summer 2003)^47^,^48^. Of these, 50 patients confirmed the consumption of fresh, raw or in-vinegar anchovies^47^. Lastly, the “Carlos III” hospital in Madrid reported approximately 30 cases (6.12 cases per 100,000 inhabitants/year, Table S9) of allergy to *Anisakis* spp. per year (González-Muñoz, unpublished data). Although, the numbers of patients reporting anisakiasis was unknown in the latter study, it was assumed, based on general agreement, that sensitization occurs via infection by live *Anisakis* spp. larvae^4^ so that the number of allergy cases indicates the incidence of anisakiasis.

The values of R and ID50 were determined using these hospital incidence data by the following two methods:

Dose-response method 1: calculation of ID50 in hospitalised anisakiasis cases assuming prior exposure to untreated anchovy meals as reported in questionnaire 1: *Determining the probability of disease*: The anisakiasis incidence, number of anisakiasis cases and duration of study for each hospital are presented in Table S9. The total number of untreated anchovy meals consumed per person each year in Spain (Amealpy, Table S9) was calculated from questionnaire 1 and used to estimate the total number of meals consumed by the catchment population of each hospital (Auntmeal, Table S9). The probability of disease from an untreated anchovy meal (Pdisease, Table S9) was determined by dividing the number of anisakiasis cases per year in that population by the total untreated anchovy meals consumed.

*Determining the Dose*: The average number of viable parasites ingested per meal was determined by running the QRA model (Dose, Table 1). This was repeated for different viabilities (100%, 50% and 10%) (Dose1, Dose0.5 and Dose0.1, Table S9).

*Determining the ID50*: Since both the dose and the probability of disease are now known, then R and ID50 can be obtained from equation 1 and 2 (Table S9). This was repeated using data from each of the four study hospitals.

Dose-response method 2: calculation of ID50 in hospitalised anisakiasis cases assuming prior exposure to untreated anchovy meals calculated using governmental estimates of anchovy consumption: *Determining the probability of disease*: This method used the reported mass of fresh whole anchovy that was used to prepare meals at home per capita (Capita, Table S9), rather than using data from the questionnaire. These data were available from MAGRAMA^31^.

For the four hospitals (“Virgen de la Salud”, “Antequera”, and “Carlos III” and “La Paz”), the consumption per capita of fresh anchovies was assumed to be the same as for the autonomous communities of Castilla la Mancha, Andalucía, and Madrid in 2013, respectively (data available from MAGRAMA^31^ and provided in Table S10).

To determine the mass of anchovy muscle eaten, the total mass of whole anchovies eaten per study population was determined (Cyear, Table S10). This was multiplied by the yield of skinless fillets obtained from European anchovy (0.51, from FAO^49^) to determine the mass of muscle eaten (Cfyear, Table S10). On average a meal consisted of 11 fillets (Anfillet, Table S10) (see section “Consumption” above). The average mass of muscle from a European anchovy was determined (8.51 g, n = 3,298) by multiplying the average total weight of the sampled anchovy (16.69 g, n = 3,298) (Table 3) by 0.51 (i.e. yield of skinless anchovy fillet^49^). Since two fillets are obtained from each fish the average fillet mass is therefore 4.25 g (Amfillet, Table S10). The average mass of a meal was calculated (Ammeal, Table S10) and from this the total number of anchovy meals consumed per year was obtained (Nmealyear, Table S10). Multiplying this by the proportion of anchovy meals that were untreated (Propunt, Tables S4 and S10), the total number of untreated anchovy meals consumed per year was obtained (Auntmeal, Table S10).

*Determining the dose and ID50:* the dose, R and ID50 were calculated as in method 1.

Dose-Response Method 3: Determination of Id50 from Questionnaire 1 Respondents Reporting Allergy. *Determining the probability of disease:* there were 716 respondents to questionnaire 1 but only 701 answered question 13S4 (Do you have allergy to *Anisakis*?). Of these, 9 confirmed they had allergy to *Anisakis* spp. It was assumed that these persons suffered an anisakiasis before the development of the allergy and that the other respondents had not suffered anisakiasis. Following correction of questionnaire 1 responses due to over-representation of respondents from Galicia the proportion of the Spanish population with allergy to *Anisakis* spp. was 0.028, and the number of allergic respondents increased to 19 persons (701 × 0.028 = 19) (Table S4).

To estimate exposure in the 701 respondents it was necessary to know the number of untreated anchovy meals they had consumed during their life. This was obtained from their age (question 2) and the number of untreated anchovy meals consumed each year by respondents. The mean age of the 701 respondents was 41.81 years. It was assumed that respondents started to eat untreated anchovy meals from the age of 18 (average 23.81 years of consumption). The number of anisakiasis per year and the incidence of the disease were finally determined using the corrected data (Casesyear and Incidence Table S9). Specifically, 1,476 untreated meals (i.e. 701 × 2.106 = 1,476) were consumed by the respondents (Auntmeal, Table S4 and S9). Finally, the probability of disease (Pdisease, Table S9) was determined as before.

*Determining the dose and ID50:* the dose, R and ID50 were calculated as in method 1.

###### ID50 Results and Discussion. 

Table 5 presents the results from the 3 different methods. Method 2 yielded higher ID50 values than method 1. This is because method 2 estimates higher numbers of untreated anchovy meals consumed by the population. ID50 values also varied depending on which hospital study was used, from 5,018 to 45,594 (with viability 100%). The ID50 estimate depends on the number of anisakiasis cases and the number of untreated anchovy meals consumed by the population. However, since the literature^4^ indicates that there is underreporting of anisakiasis and since hospitalised cases are likely to be only the most severe forms of the disease, it is likely that the value for the ID50 will be overestimated. The lower ID50 value determined in method 3 might be explained by overestimation of anisakiasis incidence from questionnaire 1. Therefore, it is possible that methods 1 and 2 overestimated the value of ID50 because they were based on hospital studies, whilst method 3 probably underestimated the ID50 because people with allergy to *Anisakis* spp. may have been more likely to respond to the questionnaire. The decrease of *Anisakis* spp. viability resulted in decreasing ID50 values, as expected since ID50 depends on the dose of exposure (which depends on viability), decreasing when the dose decreased. There is no strong evidence to adopt one method over another for selecting the ID50. Hence, all of the 9 ID50’s (with viability 100%) were used in the QRA (Table 5) utilizing the RiskDiscrete) distribution (Table 1).

#### Risk Characterization

##### Risk Calculation (Calculation of Probability of Anisakiasis Per Untreated Anchovy Meal)

The probability of an individual contracting anisakiasis from an untreated anchovy meal (Pdisunt, Table 1) was calculated using equation 1, for the 9 ID50’s described above.

##### RC Scenarios - Risk Characterization using Methods 1 and 2, Calculation of the Number of Anisakiasis Cases in 2013

Two methods were used to estimate the number of anisakiasis cases for Spain in 2013.

Method 1 (or Base scenario) used anchovy consumption data from questionnaire 1 (method 1, Table 2). The survey size, number of untreated anchovy meals consumed by questionnaire respondents and the total number of people living in Spain aged 18 and over (Popsize, Mealsyear and Spanishpop in Table 2, respectively) were used to calculate the total number of untreated anchovy meals consumed by the Spanish population in 2013 (Mealsyearspain, Table 1).

Method 2 (or Community scenario) utilised home-consumed anchovy data from MAGRAMA^31^, and questionnaire 1 information (i.e. proportion of untreated anchovy meals (i.e. 0.175, see “Propunt” in Tables S4 and S5)) for calculations (method 2, Table 2). The total number of untreated anchovy meals consumed in Spain and its autonomous communities in 2013 was calculated as follows: the total kilograms of fresh anchovy consumed at home in 2013 (Totalmass, Table S5) was available from MAGRAMA^31^. The mass of muscle of anchovies (Musclemass, Table S5) was calculated multiplying “Totalmass” by the yield of skinless fillet of anchovy (0.51)^49^. The number of fillets consumed (Numberfillets, Table S5) was calculated by dividing “Musclemass” by the average weight of an anchovy fillet (4.25 g). The number of meals (Numbermeals, Table S5) was calculated by dividing “Numberfillets” by the average number of fillets per meal (i.e. 11 fillets). Finally, the total number of untreated anchovy meals consumed at home in 2013 by the Spanish population (Numbermealsunt, Table S5) was calculated by multiplying “Numbermeals” by the proportion of untreated anchovy meals estimated from the questionnaire (0.175, “Propunt”, Tables S4 and S5).

The average numbers of anisakiasis cases in Spain (methods 1 and 2) and its autonomous communities (method 2) and the corresponding standard deviation (Anispain and Anispainsd in Table 2, respectively) were determined using the method of Lindqvist and Westwoo^27^. Briefly, the mean and the standard deviation of the probability of disease for 100,000 meals were calculated using the QRA model. The average number of cases was then obtained by multiplying the number of untreated anchovy meals consumed by the Spanish population by the mean probability of disease. The associated standard deviation, and 2.5^th^ and 97.5^th^ percentiles were calculated assuming the normal approximation of the binomial distribution (Table 2).

##### Hypothetical Scenarios

The QRA can be used to determine the change in predicted risk if any of the model parameters change. This procedure was tested in two ways which could result in a behaviour change in the way the anchovy are prepared. The first was to determine the increase in risk caused by *post-mortem* migration of *Anisakis* spp. from the fish viscera to the muscle (worst case scenario). The second was a risk mitigation scenario (hence targeted to reduce risk) that involved an educational campaign.

Worst case scenario - *post-mortem* migration of *anisakis* spp. from fish viscera to the muscle. *Post-mortem* migration of *Anisakis* spp. from viscera to muscle has been reported in European anchovy^19^,^20^. The value chain of European anchovy (Fig. 1) starts with fish being caught at sea, stored in ice and landed within one to two days. Then, the anchovies are transported in boxes with ice to retail (supermarket, fish market, etc.), where they are purchased by the consumer who carries the fish in plastic bags to home at ambient temperature where they are then refrigerated. Only then are the fish cleaned and eviscerated (usually no evisceration occurs before this step). This usually takes place on the day of purchase. Thus, migration of *Anisakis* spp. into muscle can occur at any point prior to evisceration.

The QRA model was modified to simulate the worst case scenario (i.e. anisakiasis caused by inadequately stored anchovies that allow *Anisakis* spp. *post-mortem* migration from viscera to muscle) as follows:

Cipriani *et al*.^19^ found that in batches of 100 anchovy immediately frozen after capture, 20% of the fish had *Anisakis* spp. in the muscle with a total of 28 larvae. However, if the anchovies were left for 72 hours at 7 °C (temperature typical of a fridge in Spanish household^50^) that muscle prevalence increased to 57% with 117 larvae being present. Additional parameters were incorporated into the QRA model to determine changes in prevalence, number of parasites consumed per meal, probability of anisakiasis and finally the number of anisakiasis cases in Spain when this worst case scenario is considered, as follows: The prevalence of the worst case scenario (Ptotalmigration) was determined by multiplying the parameter “Pselected” (Table 1) by the increase in prevalence (i.e. 57/20). The number of infected fish per meal (Nfishinfmigration) was determined as the parameter “Nfishinf” (Table 1), using the RiskBinomial distribution (i.e. RiskBinomial (Nfish,Ptotalmigration)). The number of ingested *Anisakis* spp. per meal (Nparamigration) was determined similarly to the variable “Nparasite” (Table 1), but multiplying the intensity of infection (i.e. NparasitesArea1, 2 and 3, Table 1) by “Nfishinfmigration”, and by the increase in the number of *Anisakis* spp. per fish (i.e. 117/28). The probability of anisakiasis (Pdisuntmigration) was determined as the parameter “Pdisunt” (i.e. 1-EXP(-R*Nparamigration). Finally, the number of anisakiasis predicted was calculated as the parameter “Anispain” (Table 2) (i.e. Mealsyearspain*Pdisuntmigration).

Risk mitigation scenario – public health education campaign: Del Rey Moreno *et al*.^48^ reported that in Antequera, human anisakiasis was reduced from 10–16 to 0–2 cases per year, following a public health education campaign and implementation of RD 1420/2006^32^. This recommended consumers to freeze fish prior to raw consumption and also obliged sellers to freeze fish that would be served raw or partially raw. The QRA model was modified to simulate this education campaign. The number of untreated meals consumed by the Spanish population after the education campaign (“Mealsmitigation”) was calculated multiplying 2/10 (i.e. most conservative ratio of number of cases post-intervention to those prior to the intervention) by the total untreated meals eaten by the Spanish population before the education campaign (Mealsyearspain, Table 1). Finally, the effect of the mitigation strategy on the number of anisakiasis was determined (i.e. Mealsmitigation*Pdisunt).

### Results

#### Base Results of the QRA Model

##### Number of Viable Anisakis Spp. Consumed Per Untreated Anchovy Meal (i.e. Dose of Exposure)

**Figure 2:**
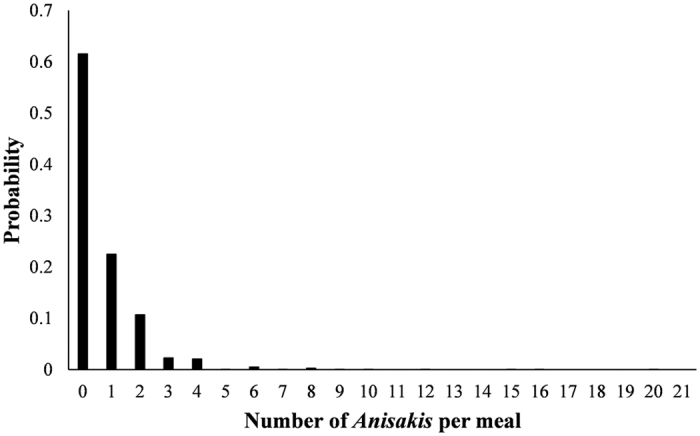
Probability density function of the number of viable *Anisakis* spp. consumed per untreated anchovy meal with 100% viability (mean 0.66, SD 1.15).

The QRA model estimated the average number of viable *Anisakis* spp. consumed per untreated anchovy meal to be 0.66 with standard deviation (SD) 1.15. This distribution was right-skewed (Fig. 2), with 38.5% of meals containing at least one viable *Anisakis* spp.

##### Probability of Anisakiasis (Disease) Per Untreated Anchovy Meal

**Figure 3:**
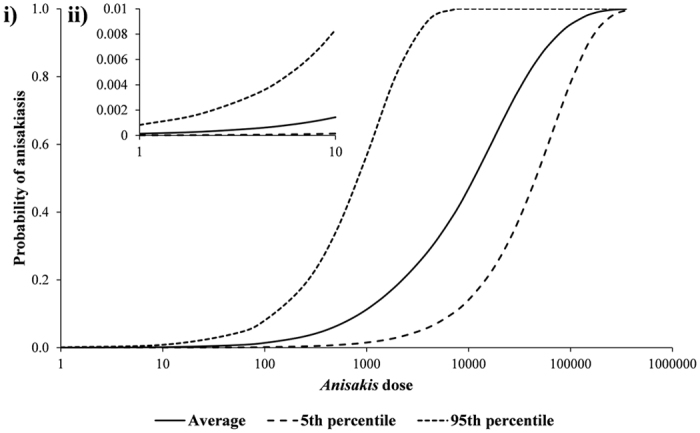
(i) Dose response for *Anisakis* spp. (ID50, mean 17,000, SD 14,000), (ii) inset of dose response at higher resolution for low doses (i.e. exposure between 1 and 10 parasites).

**Figure 4:**
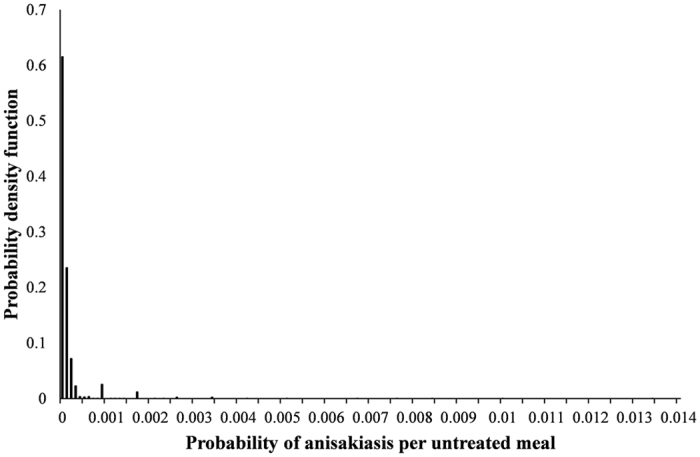
Probability density function of the probability of disease (anisakiasis) per untreated anchovy meal (mean 9.56 × 10^−5^, SD 3.68 × 10^−4^).

The dose response for *Anisakis* spp. generated by applying equal weighting to the nine ID50 estimates is presented in Fig. 3. The resulting mean ID50 was 17,000 with SD 14,000. Using this dose response, the probability density function of the probability of disease per untreated anchovy meal was obtained (Fig. 4). From this the average probability of humans contracting anisakiasis per untreated anchovy meal was found to be 9.56 × 10^−5^ with SD of 3.68 × 10^−4^. Thus, on average for every 10,456 meals consumed there was 1 anisakiasis case (since 1/10,456 is 9.56 × 10^−5^).

#### Risk Characterization Results - Methods 1 (Base Scenario) and 2 (Community Scenario)

The QRA model predicted an average annual number of anisakiasis cases of approximately 7,700 ± 90 (SD), 2.5^th^ and 97.5^th^ percentiles [7,560–7,850] for method 1 and 8,320 ± 90 (SD), 2.5^th^ and 97.5^th^ percentiles [8,170–8,470] for method 2 in Spain (Table 6). The anisakiasis incidence varied between 18 and 20 cases per 100,000 inhabitants/year for method 2 and method 1, respectively.

The average number of anisakiasis cases was also determined for Spain and its Spanish autonomous communities using method 2 (Table 7). The largest number of anisakiasis cases was estimated to occur in Andalucía (n = 2,220 ± 50 (SD)), followed by Madrid (n = 1,280 ± 40 (SD)). In terms of anisakiasis incidence, Cantabria (35 cases per 100,000 inhabitants/year) followed by País Vasco (31 cases per 100,000 inhabitants/year) had the highest values.

#### Hypothetical Scenarios Results (Estimated using Risk Characterization Method 1)

##### Worst Case Scenario – Post-Mortem Migration of Anisakis Spp. from Fish Viscera to the Muscle

The QRA model predicted an annual average number of anisakiasis cases when *Anisakis* spp. migration was considered of approximately 91,100 ± 300 (SD) in Spain (see “worst case scenario” in Table 6). This resulted in an increase of >1,000% in the number of anisakiasis predicted by the base scenario. The base scenario assumes that there is no migration of *Anisakis* spp. larvae from the viscera to the muscle.

##### Risk Mitigation Scenario – Public Health Education Campaign

The QRA model estimated an annual average number of anisakiasis cases after an education campaign of approximately 1,540 ± 40 (SD) in Spain (Table 6). This was a reduction of 80% compared with the Base scenario which is as expected. However, the responses to the questionnaire suggest that the scenario is more complex due to human behaviour. Notably, it was found that among those respondents (n = 46) who consumed untreated anchovy meals, 89% (41 out of 46 respondents) answered correctly that marinating or smoking the fish does not prevent *Anisakis* spp. (question 20 A). Moreover, 89% (40 out of 46 respondents) answered correctly that freezing prevents disease (question 20 A) but that they continue to eat raw untreated meals.

## General Discussion

### Estimating the number of anisakiasis cases in Spain

The results generated by the QRA suggest that Spain has between 7,700 and 8,320 anisakiasis cases annually. Since these data are predominantly based on hospitalised cases to determine the ID50, it is likely that the actual number of cases across Spain will be higher, when mild infections are included. The QRA estimates apparently suggest a higher incidence of anisakiasis in Spain than previously reported for Japan (about 2,000–3,000 cases per year^4^,^34^), but it has to be borne in mind that the methodology was different (Japanese estimations are based on annual diagnosed cases and it is likely that the efficacy of the reporting systems are different^4^,^34^) and the comparison is therefore not valid. However, it is clear that published estimates of 500 anisakiasis cases in Europe per year^51^ or 20 cases on average per European country per year^14^ are very likely to be a considerable underestimation.

It is frequently claimed that the number of anisakiasis cases is underdiagnosed and misdiagnosed for three reasons. First, due to the non-specific symptoms of the disease and lack of clinical investigation^4^, second confounding with other gastrointestinal conditions^4^,^10^,^11^ and third it may remain undiagnosed if ingestion of raw or undercooked fish is not considered during anamnesis^48^. Although symptomatic anisakiasis has been reported, asymptomatic intestinal anisakiasis^5^ or symptomless clinical presentation^9^ have also been observed. Toro *et al*.^11^ suggested that human anisakiasis might be underdiagnosed in Spain because only the most severe cases that required intervention are being diagnosed. BIOHAZ^4^ suggested that the 96 gastroallergic anisakiasis cases reported from the “La Paz” area of Madrid^40^ could be an underestimation since many patients with mild symptoms were unlikely to seek medical attention. Hence, in reality it appears that the true burden of disease in the Spanish population is likely to exceed the estimates based on the risk assessment model.

### Comparison of the two Risk Characterization methods and potential biases

The total number of anisakiasis estimated in Spain in 2013 was calculated by two RC methods. The total number of untreated anchovy meals eaten annually as estimated by RC method 2 is more than 6 million greater than estimated by RC method 1, resulting in approximately 7% more anisakiasis cases. This difference reflects the different source of data used, namely questionnaires for RC method 1 and governmental estimates for RC method 2, as well as the methodology used during calculations of the number of untreated meals consumed by the population. In addition, RC method 1 assumed that only adults > 18 years old consume untreated anchovy meals. Hence, RC method 1 may underestimate the total number of untreated meals consumed by the population and, therefore, the total annual number of anisakiasis cases occurring in Spain for this reason, since people under 18 may also consume such meals.

Correcting for the over-representation of respondents from Galicia in questionnaire 1 made a significant difference to the number of cases estimated by RC method 1. When the model is run without the correction it predicted 4,650 cases (data not presented) instead of 7,700. This is explained by the difference in the number of untreated anchovy meals consumed which is lower in Galicia compared with the rest of Spain. These findings emphasise the importance of having a representative sample of the Spanish population when conducting a questionnaire survey.

The regional differences found in response to questionnaire 1 are supported by published information. In mainland Spain, Galicia was estimated to be the region with the lowest anisakiasis incidence (5 cases per 100,000 inhabitants/year, Table 7), and is the community with lowest consumption of fresh anchovies at home per capita (i.e. 0.31 kg per year)^31^. Valiñas *et al*.^42^ reported that 12.8% (n = 13/101) of Galician individuals (control subjects seronegative to *Anisakis*) prepared untreated raw anchovies at home. This figure is over three times higher than that found in the current study in Galicia (3.8%, n = 17/447) but similar to that found in the rest of Spain (10.6%, 28/263). It is unclear which study is the most representative, but the current work does have a larger sample size.

The ID50 for *Anisakis* spp. was determined using 3 dose-response methods. Method 1 and 2 may have overestimated the value of the ID50 because they were based on hospital studies (i.e. it is likely that only the most severe cases were diagnosed). Whilst method 3 may have underestimated the ID50 because it is based on respondents reporting allergy to *Anisakis* spp. in questionnaire 1 and it is possible that individuals with allergy were more likely to respond to the questionnaire. It is also worth noting that when correcting for the over-representation of Galicia in questionnaire 1, the final dose response did not change significantly (corrected ID50 was 17,000 with SD 14,000 and uncorrected ID50 was 15,000, SD 14,000).

Questionnaire 1 had 716 respondents which is only 0.002% of the population of Spain aged 18 and over. Bootstrapping of the raw questionnaire responses was used (results not presented) to incorporate variation (i.e. generate distributions in the number of responses by community and untreated anchovy meals consumed)^52^. This leads to a change in the variance but not a change in the mean values of the variables. Re-running the model with these distributions rather than fixed values as done in the QRA model provided virtually identical results for the number of cases and associated standard deviations for RC methods 1 and 2.

It was assumed that a respondent ate 1, 12 or 52 meals per year when the respondent answered annually, monthly or weekly anchovy consumption when completing the relevant questions of questionnaire 1 and 2. It is likely that respondents may not have remembered exactly what they had eaten in the previous year and this categorisation of responses would have introduced some variation into these data.

### Anchovy is the main fish species causing anisakiasis

The QRA model was built assuming that all anisakiasis cases predicted in Spain in 2013 were caused by home-prepared untreated anchovy meals. While other fish species and recipes have also been implicated, the great majority of patients for which data is available had eaten anchovies. For instance, in the “Antequera” hospital study, 50 of 52 anisakiasis patients consumed fresh, raw or in vinegar anchovies prior to falling ill, but one patient consumed raw sardine (and there was no food consumption information for the remaining patient)^47^,^48^. Also 18 of 96 gastroallergic anisakiasis cases in the “La Paz” hospital study consumed fish species other than anchovy (9 patients consumed undercooked hake, 2 patients consumed raw cod and 7 patients consumed other cooked fish) prior to falling ill^40^. In the Repiso Ortega *et al*.^46^ (i.e. “Virgen de la Salud” hospital study) all 25 anisakiasis patients had a history of raw anchovy ingestion. Thus, raw or marinated anchovies appear to be the dominant vehicle of anisakiasis in Spain but it should be remembered that other fish species (and recipes) can contribute to the disease burden in humans.

### Home-prepared raw and marinated anchovies are the main fish specialties causing anisakiasis

It was assumed that untreated home-prepared raw and marinated anchovies was the fish specialty involved in parasite transmission to humans. However, raw fish ingestion at restaurants and bars may also be implicated as a vehicle of disease, especially for the hospital studies (i.e. “La Paz”, “Virgen de la Salud” and “Antequera”) performed before the implementation of the Royal Decree RD 1420/2006^32^ which requires freezing prior to raw consumption. It was therefore possible that the estimated ID50 values for the latter hospital studies could be low due to underestimation of the number of untreated anchovy meals consumed by the catchment population of each hospital. However, considering that anchovies consumed in restaurants and bars are often of industrial origin (i.e. prepared from frozen fish)^42^, and since the Carlos III hospital study was performed after the implementation of RD 1420/2006^32^ (“Carlos III” ID50 values were not the highest), this may not be a significant issue.

### Viability of *Anisakis* spp. in untreated anchovy meals and their inactivation in the human stomach

It has been demonstrated that the marinating process does not inactivate all *Anisakis* spp. larvae present^4^. However, the action of ingredients (e.g. vinegar, lemon, olive oil, garlic, parsley, salt, etc., usually present in some raw or marinated specialties) and storage time may reduce the viability or alternatively reduce the pathogenic potential of larvae. *Anisakis* spp. can survive the traditional Spanish marinating procedure, even though its survival can be compromised by increasing the concentration of salt, acetic acid and storage time in brine^44^. *In vitro* studies have demonstrated that saline extracts from garlic can be destructive to *Anisakis* spp.^53^. The QRA model was run considering viability of 100%, even though viability could be lower, and ID50 values for viabilities of 50% and 10% were therefore also generated.

### Implementation and validity of the exponential dose response model for *Anisakis* spp

It has been reported that infection with a single parasite may cause severe health problems that may require surgical treatment^40^,^46^,^48^ and hence a single hit dose-response, where one organism has a finite probability of causing disease, is appropriate. The two main forms of such a model are the exponential, that is used here, and the beta-Poisson^45^. The beta-Poisson model incorporates heterogeneity in both the parasite and the host, whereas the exponential model assumes that all parasites and hosts are identical. The ID50´s were determined by estimating exposure across a population that had a specific number of cases reported to the local hospital or from questionnaire 1 respondents reporting allergy to *Anisakis* spp. These are novel approaches but could be biased because of heterogeneity in host immune response and heterogeneity in *Anisakis* spp. pathogenicity (see next section for further discussion). Since four different hospital studies were included, it is anticipated that this will incorporate some of the uncertainty in the data from across Spain but it should be noted that these hospitals only serve 3% of the total population (Spanish Statistical Office - available at: http://www.ine.es/accessed 3^rd^ May 2016). It is also worth noting that these methods of estimating the dose-response are advantageous ethically as they provide an alternative to carrying out animal and human studies.

### Pathogenicity of *Anisakis* spp. species and susceptibility of humans

The QRA model was performed considering *Anisakis* spp. as a single pathogen even if *A. pegreffii* and presumably *A. simplex* s.s. have been identified in anchovies^18^,^19^,^20^. Some studies have suggested differences between *A. pegreffii and A. simplex* s.s. in terms of immunopathogenicity capacity against humans^51^,^54^,^55^. Humans may also have different susceptibilities to these zoonotic nematodes^4^. Caballero *et al*.^56^ found differences in clinical and immunological symptoms between Italian and Spanish *Anisakis* spp. allergic patients. Moreover, the presence of high risk population groups (e.g. elderly population) and major risk factors (e.g. raw fish consumption) for anisakiasis is also important. Further improvement of the model could be implemented if heterogeneity of human host and parasite species is confirmed and data become available.

### Sensitization and allergy to *Anisakis* spp

Allergy to *Anisakis* spp. is relatively common in some Spanish regions^4^,^6^ and subclinical sensitization (i.e. *Anisakis*-specific IgE detection in individuals who do not show allergic manifestations) may also occur^41^,^42^,^57^,^58^,^59^. For instance, Del Rey Moreno *et al*.^57^ reported seroprevalence to *A. simplex* of 22.1% (n = 17 out of 77 random blood donors) tested by CAP-FEIA in a healthy Spanish population (“Antequera” hospital region). Other studies in healthy populations showed seroprevalence ranging between 6.6 and 27.5%^41^,^57^. Valiñas *et al*.^42^ reported seroprevalence of 0.4% (n = 12 out of 2801 random blood donors from Galicia) tested by an antigen-capture ELISA method, and suggested that more than 150,000 individuals may have IgE sensitization to *Anisakis* spp. in Spain. It is generally considered that sensitization with living *Anisakis* spp. larvae is required prior to development of the clinical allergic responses^4^,^7^. Therefore, these data suggest that a considerable proportion of the Spanish population is sensitized by *Anisakis* spp. infection at some point in their life, even though the disease (anisakiasis) was not diagnosed or asymptomatic, and therefore remains underreported. However, it has been suggested that ingestion (and inhalation) of dead larvae or their related allergens might also initiate allergic problems^4^. In addition, cross-reactivity with other parasites, insects and shellfish has also been suggested^4^, so the allergy debate is still open.

A number of studies have suggested that differences in seroprevalence to *Anisakis* spp. in different Spanish regions can be explained by differences in raw fish consumption habits of the population^41^,^58^,^59^. The results presented here are in accordance with this hypothesis, since the communities (Andalucía and Madrid) with the highest consumption of untreated anchovy meals, presented the highest numbers of anisakiasis cases, whilst the communities (Cantabria and País Vasco) with highest consumption of untreated meals per capita, presented the highest incidence of disease. In addition, a multicentre study found that 8% of Spanish patients presented with some form of allergic reaction, of which 38.1% were sensitized and 19.2% were allergic to *Anisakis* spp., had reported consumption of non-cooked fish at least once a week, with anchovies in vinegar being the most frequent meal^59^.

### *Post-mortem* migration of *Anisakis* spp. from fish viscera to the muscle

The QRA results showed that the number of parasites consumed per meal and the total number of anisakiasis cases would increase considerably following inadequate storage of whole anchovies at 7 °C during 72 hours pre-evisceration. Typical temperatures of 7 °C have been reported in food stored in Spanish domestic refrigerators^50^. This enables migration of *Anisakis* spp. from fish viscera to the muscle if the fish are stored whole. Cipriani *et al*.^19^ reported no significant increase in the *A. pegreffii* infection rates in anchovy muscle when fish were refrigerated at 2 °C when examined 24, 48 and 72 h after capture in the Mediterranean Sea. However, significant increases in infection rates in muscle were observed when fish were refrigerated at 5 °C and 7 °C at all storage times. In addition, Šimat *et al*.^20^ similarly studied *post-mortem A. pegreffii* migration from anchovy viscera to muscle when fish were refrigerated at 0 °C and 4 °C after zero, three, five and seven days after capture in the Mediterranean Sea. *Post-mortem* migration of *A. pegreffii* was observed in anchovy muscle refrigerated at 0 °C and 4 °C after five and three days post capture, respectively^20^. Thus, temperatures of 2 °C appears therefore to be sufficient through the anchovy value chain prior to retail sale to prevent *post-mortem* migration of *A. pegreffii*. Further work is required to determine if higher temperatures such as 3 °C or 4 °C would also be sufficient to prevent *A. pegreffii post-mortem* migration for at least 48 hours (anchovies should be at retail for less than two days to ensure organoleptic quality). Further work is also needed to determine what temperatures and storage times would prevent *A. simplex* s.s. *post-mortem* migration in European anchovies from Atlantic waters. It is also important for consumers to know that fresh anchovies should be eviscerated as soon as possible after purchase and frozen. It is likely that this could be achieved through an education campaign.

### Risk mitigation strategies

The QRA simulation demonstrates the trivial result that if an education campaign results in an 80% increase in the number of anchovy meals that are frozen then this results in an 80% reduction in the incidence of anisakiasis in the human population. However, the finding from questionnaire 1 that 89% of those consuming untreated anchovy meals knew that freezing was required to prevent anisakiasis but currently do not carry this out suggests that it is important to target this group of individuals. Further, it is necessary to understand why they do not freeze anchovy and what motivation is required for them to change their behaviour. This should be addressed in order to inform targeted public health education campaigns in Spain. Further, an educational campaign using the media (e.g. press, television, etc.) may be best targeted in the spring and summer months when anchovies in vinegar are most frequently consumed, and especially in those communities with higher numbers of anisakiasis cases (Andalucía and Madrid) and higher incidence of the disease (Cantabria and País Vasco). Other mitigation strategies, such as removal of anchovy viscera by retailers to prevent parasite migration may also reduce disease incidence and it is also important to find out how retailers and/or consumers can be persuaded to do this^14^.

### Extending the QRA method to other countries and other parasites

The QRA model focussed on the anchovy value chain from the sea to the consumer. The model has the potential to be applied to other countries that also have anisakiasis (e.g. Japan and Italy). The model could be parameterised to include other fish species (e.g. hake, herring) and other methods of preparing fish (e.g. under or lightly cooked fish). Moreover, anisakidosis caused by other zoonotic anisakids (e.g. *Pseudoterranova* spp., *Contracaecum* spp.) or other fish-borne parasitic zoonoses (e.g. ophistorchiasis, clonorchiasis, intestinal trematodiasis and diphyllobothriasis) may be assessed for risk. In the first instance, the *Anisakis* spp. dose-response model in this paper could be used as a surrogate as has been done previously (e.g. using *Shigella* spp. dose response for *E. coli* O157^21^). However, if data are available then dose response models for these other parasites could also be developed.

In the QRA model the inclusion of economic data on fish catches and imports, fish parasite abundance surveys, questionnaire information on consumption habits and preferences can be updated over time. This will enable temporally dynamic estimates of the disease burden of this zoonosis to be performed and also identify which factors are important in its ongoing emergence. These techniques are readily applicable to a number of other infectious diseases including gastrointestinal pathogens^21^,^25^,^26^,^27^.

Finally, the results of the QRA model are only accurate to the extent that the input data are valid and the model variables represent the process. The results seem plausible, even though they were estimated based on a number of assumptions that are described above. Further research is recommended to validate the findings and this could involve an intensive study of consumption patterns and epidemiological investigations in representative communities within Spain. The model incorporates the variation in the datasets that were obtained. However, there are uncertainties in whether some of these data are properly representative across Spain. Further work needs to be done to include this uncertainty into the model (e.g. via a second-order Monte Carlo model) to determine the effect this has on model outputs and identify which model parameters are most uncertain.

## Conclusions

This is the first time that a QRA study of anisakiasis caused by fish meals has been performed and it integrates data obtained from natural and social science methods. The results indicate that anisakiasis is a highly underreported disease (e.g. by misdiagnosis, undiagnosed and unreported cases). The annual number of anisakiasis cases that required medical attention in Spain is estimated to be between 7,700 and 8,320 using the two risk characterisation methods, with 42% occurring in the Spanish communities of Andalucía and Madrid (RC method 2). These results suggest that Spain has a high anisakiasis incidence, compared with other countries, but this needs verified by implementation of comparable methods of analysis between countries. The dose response for *Anisakis* spp. and corresponding ID50 (mean 17,000, SD 14,000) was reported for the first time. On average, the QRA estimates that 0.66 *Anisakis* spp. are consumed per raw or marinated anchovy meal in Spain.

This study makes use of the ecological information that is known about *Anisakis* spp., the anchovy value chain, as well as human behaviour, and puts this into the context of risk and what can be done to mitigate the disease. In addition, the methods have the potential to be applied to other countries that also have anisakiasis, fish-borne zoonosis and different cooking preparations. The results are of relevance to industry, medical practitioners and consumers, and can be used to inform policy (e.g. by food safety authorities) and to reduce risk of disease in two ways. Firstly, by highlighting the need for adequate cold storage of anchovies, early evisceration, and parasite monitoring (e.g. improved protocols and technology for parasite detection in fishery products) to control the pathogen along the value chain, and secondly, identifying the importance of changing consumer habits, particularly those who currently eat untreated anchovy meals, by education campaigns to encourage freezing of fish prior to consumption. The efficacy of these strategies can be monitored by observing changes in disease incidence through improved reporting and by monitoring behavioural changes in anchovy preparation methods and consumption preferences by questionnaire. Then it will be possible to make progress in reducing the disease burden of this emerging zoonosis.

## Additional Information

**How to cite this article:** Bao, M. *et al*. Assessing the risk of an emerging zoonosis of worldwide concern: anisakiasis. *Sci. Rep.* **7**, 43699; doi: 10.1038/srep43699 (2017).

**Publisher's note:** Springer Nature remains neutral with regard to jurisdictional claims in published maps and institutional affiliations.

## Supplementary Material

Supplementary Information

## Figures and Tables

**Table 1:** QRA model variables used to determine the probability of anisakiasis per untreated anchovy meal.

Short description	Section in paper	Variable	Units	Distribution, fixed value or selection	Data source
**EXPOSURE ASSESSMENT**					
Selecting Sea area	Fishing area	Area	Number	RiskDiscrete({1,2,3},{.55,.43,.03})	Table S2
Prevalence in muscle fishing area 1	Prevalence & Intensity	PArea1	Proportion	137/2,913 (approximately 0.05)	Table 3
Prevalence in muscle fishing area 2	Prevalence & Intensity	PArea2	Proportion	136/886 (approximately 0.15)	Table 3
Prevalence in muscle fishing area 3	Prevalence & Intensity	PArea3	Proportion	Fixed value at 0.1313	Table 3
Prevalence in muscle of “Area” selected	Prevalence & Intensity	Pselected	Proportion	By IFs from “Area” selected	
Number of *Anisakis* in flesh per infected anchovy from area 1	Prevalence & Intensity	NparasitesArea1	Number	RiskDiscrete({1,2,3,4},{.825,.139,.029,.007})	Table 4
Number of *Anisakis* in flesh per infected anchovy from area 2	Prevalence & Intensity	NparasitesArea2	Number	RiskDiscrete({1,2,3,4},{.801,.147,.022,.029})	Table 4
Number of *Anisakis* in flesh per infected anchovy from area 3	Prevalence & Intensity	NparasitesArea3	Number	RiskDiscrete({1,2,3,4},{.808,.115,.058,.019})	Table 4
Number of fillets in a meal	Consumption	Nfillet	Number	RiskDiscrete({1:39},{0.00462: 0.00003})	Anchovy meal size sub-model; Table S8
Number of fish in the meal	Consumption	Nfish	Number	Obtained from Nfillet but corrects for 2 fillets per fish	
Number of fish with at least one parasite present	Consumption	Nfishinf	Number	RiskBinomial(Nfish,Pselected)	
Number of *Anisakis* in meal	Consumption	Nparasite	Number	IF(Area = 1,NparasitesArea1)*Nfishinf Select NParasitesArea1 if Area1, do likewise if Area2 or Area3.	
Proportion of *Anisakis* that are viable	Viability	Propviable	Proportion	Set at 1 (100% viable). Also, 0.5 and 0.1.	
Number of viable *Anisakis* in meal	Viability	Dose	Number	ROUND(Nparasite*Propviable,0)	
**HAZARD CHARACTERIZATION**					
Dose of parasite required to produce disease in 50 percent of subjects	Hazard characterization	ID50	Number	RiskDiscrete({5018,24897,8153,15737,10439,45594,10276,32737,828},{.11,.11,.11,.11,.11,.11,.11,.11,.11})	Table 5
Probability of one parasite surviving in host to cause disease	Hazard characterization	R	Number	R = -Ln(0.5)/ID50	
**RISK CHARACTERIZATION**					
Probability of disease per untreated anchovy meal	Risk calculation	Pdisunt		1-EXP(-R*Dose)	

**Table 2:** QRA Risk Characterization model variables to determine the number of anisakiasis in Spain (method 1 or Base scenario) and in Spain and its Autonomous Communities (method 2 or Community scenario).

RISK CHARACTERIZATION SCENARIOS					
METHOD 1 (Base scenario) estimates the number of anisakiasis per year in Spain using anchovy consumption data from questionnaire 1					
Short description	Section in paper	Variable	Units	Fixed value	Data source
Number of questionnaire respondents	RC Scenarios	Popsize	Number	716	Questionnaire 1
Untreated anchovy meals eaten by questionnaire respondents per year	RC Scenarios	Mealsyear	Number	1,508	Table S4
Spanish population aged 18 and over (at 01_07_13)	RC Scenarios	Spanishpop	Number	38,241,133	INE^a^
Untreated anchovy meals eaten by Spanish population per year	RC Scenarios	Mealsyearspain	Number	Mealsyear*Spanishpop/Popsize = 80,541,619	
Mean number of anisakiasis in Spain per year caused by untreated anchovy meals	RC Scenarios	Anispain	Number	Mealsyearspain*Pdisunt^b^	
Standard deviation of Anispain	RC Scenarios	Anispainsd	Number	(Mealsyearspain*Pdisunt*(1-Pdisunt))^0.5	
Normal approximation of the binomial distribution				RiskNormal(Anispain, Anispainsd)	
**METHOD 2 (Community scenario) utilises anchovy consumption data from MAGRAMA (2016), and questionnaire 1 information (i.e. proportion of untreated anchovy meals, see “Propunt” in Table S4) for calculations**					
**Short description**	**Section in paper**	**Variable**	**Units**	**Fixed value**	**Data source**
Untreated anchovy meals eaten in Spain and its autonomous communities per year	RC Scenarios	Nmunt	Number		Values of “Nmunt” in Table S5
Mean number of anisakiasis in Spain and its autonomous communities per year caused by untreated anchovy meals	RC Scenarios	Anispain2	Number	Nmunt*Pdisunt	
Standard deviation of Anispain2	RC Scenarios	Anispain2sd	Number	(Nmunt*Pdisunt*(1-Pdisunt))^0.5^	
Normal approximation of the binomial distribution			Number	RiskNormal(Anispain2,Anispain2sd)	

^a^INE (Spanish Statistical Office). Available at: http://www.ine.es/.

^b^Pdisunt: Probability of disease per untreated anchovy meal, see Table 1 at Risk Characterization.

**Table 3:** Descriptors of *Anisakis* spp. infection in European anchovy and fish biometrics.

Fishing area (n)	Year	TW ± SD (range)	Ninf	Ptotal	Im (range)	Source of data
1: FAO 37 (2,913)	2013, 2014, 2015	14.90 ± 4.16 (4–31)**	137	5%	1.22 (1–4)	PARASITE project*
2: FAO 27 (886)	2014, 2015	21.54 ± 4.83 (12–42)	136	15%	1.28 (1–4)	PARASITE project*
3: FAO 34 (396)	1998, 1999	Not available	52	13%	1.28 (1–4)	Estimated from Rello *et al*.^18^

Fishing area (n), area of capture and number of anchovies analysed; year; TW ± SD (range), total mean weight and standard deviation (minimum – maximum) (g); Ninf, number of anchovies with infection in muscle; Ptotal, prevalence of infection in muscle; Im (range), mean intensity of infection in muscle (minimum – maximum).

^*^Data generated within the EU FP7 PARASITE project (GA no. 312068).

^**^The TW of 501 anchovies was not available and therefore not included in calculations.

**Table 4:** Distribution of the number of parasites in the muscle of infected anchovies by fishing area. N, number of *Anisakis* spp. in muscle per infected anchovy; Frequency, number of fish infected; Proportion, proportion of fish with *Anisakis* spp. infection.

	Fishing area 1	Fishing area 2	Fishing area 3
N	Frequency (proportion)	Frequency (proportion)	Frequency (proportion)
1	113 (0.825)	109 (0.801)	42 (0.808)
2	19 (0.139)	20 (0.147)	6 (0.115)
3	4 (0.029)	3 (0.022)	3 (0.058)
4	1 (0.007)	4 (0.029)	1 (0.019)
Total	137 (1)	136 (1)	52 (1)

**Table 5:** Dose response ID50 values determined for hospital populations (H1, “La Paz”; H2, “Virgen de la Salud”; H3, “Antequera” and H4, “Carlos III”) using dose response methods 1 and 2, and for the questionnaire population (Qaire) using method 3. Results for different viabilities of *Anisakis* spp. are also presented (100% (Base scenario), 50% and 10%).

Viability	Method 1				Method 2				Method 3
	H1	H2	H3	H4	H1	H2	H3	H4	Qaire
100%	5,018	24,897	8,153	15,737	10,439	45,594	10,276	32,737	828
50%	3,421	16,975	5,559	10,730	7,117	31,087	7,006	22,321	565
10%	76	377	124	238	158	691	156	496	13

**Table 6:** QRA model estimated anisakiasis cases and incidence of disease in Spain during 2013.

Risk characterization	Anisakiasis ± SD	Percentage^a^	Incidence (cases per 100,000 inhabitants/year)
RC method 1 (Base scenario)	7,700 ± 90		20^b^
RC method 2 (Community scenario)	8,320 ± 90	7%	18^c^
Worst case scenario (migration)	91,100 ± 300	>1,000%	
Risk mitigation scenario (education)	1,540 ± 40	−80%	

^a^Percentage increase/decrease compared to risk characterization method 1.

^b^Incidence of anisakiasis for Spanish population aged 18 and over (i.e. 38,241,133 inhabitants) (at 01_07_13) (Source: INE (Spanish Statistical Office) available from: http://www.ine.es/).

^c^Incidence of anisakiasis in the whole Spanish population (i.e. 46,593,236 inhabitants) (at 01_07_13) (Source: INE (Spanish Statistical Office) available from: http://www.ine.es/).

**Table 7:** Estimated cases of anisakiasis and its incidence (i.e. cases per 100,000 inhabitants/year) in Spain and its autonomous communities in 2013 using risk characterization method 2 (Community scenario).

Community	Anisakiasis ± SD	Incidence	Community	Anisakiasis ± SD	Incidence
Total Spain	8,320 ± 90	18	Cataluña	1,140 ± 30	15
Andalucía	2,220 ± 50	27	Extremadura	160 ± 10	14
Aragón	190 ± 10	14	Galicia	160 ± 10	6
Asturias	190 ± 10	18	La Rioja	70 ± 10	21
Baleares	90 ± 10	8	Madrid	1,280 ± 40	20
Canarias	20 ± 4	1	Murcia	210 ± 10	14
Cantabria	210 ± 10	35	Navarra	110 ± 10	16
Castilla la Mancha	430 ± 20	21	País Vasco	670 ± 30	31
Castilla y León	460 ± 20	18	Valencia	730 ± 30	15
