# Supplementary material for: Assessing the risk of an emerging zoonosis of worldwide concern: anisakiasis
Source: Sci Rep. 2017 Mar 13;7:43699. doi: 10.1038/srep43699 (PMC5347442; doi:10.1038/srep43699)
Supplement: Supplementary Information [file srep43699-s1.pdf]

Supplementary information

Assessing the risk of an emerging zoonosis of worldwide concern: anisakiasis

Miguel Bao, Graham J. Pierce, Santiago Pascual, Miguel González-Muñoz, Simonetta Mattiucci, Ivona Mladineo, Paolo Cipriani, Ivana Bušelić & Norval J.C. Strachan

Models

The quantitative risk assessment model (QRA model) and the "anchovy meal-size" sub-model are available by request from the authors.

Current file

In the current file are supplementary tables S1-S10, questionnaire 1 (Spanish version) and questionnaire 2. In addition, provided is an English version of questionnaire 1 disseminated in Scotland. Please, note that there are some minor differences in the questionnaires because some questions were adapted to each country.

References

1. FAO. Fishery Statistical Collections - Global Production Statistics. (2016). Available at <http://www.fao.org/fishery/statistics/global-capture-production/en>. (Accessed: 11th March 2016).
2. MAGRAMA. Database consumption in households. (2016). Available at <http://www.magrama.gob.es/es/alimentacion/temas/consumo-y-comercializacion-y-distribucion-alimentaria/panel-de-consumo-alimentario/base-de-datos-de-consumo-en-hogares/consulta10.asp>. (Accessed: 11th March 2016).
3. FAO. *Yield and nutritional value of the commercially more important fish species.* (1989). Available at <http://www.fao.org/docrep/003/t0219e/t0219e00.htm>. (Accessed: 11th March 2016).

Table S1. Global production statistics (production) of European anchovy from supplier countries in 2013 (FAO)[1]. Total imports of fresh anchovy from supplier countries ("Ministerio de Agricultura, Alimentación y Medio Ambiente – Spanish government", MAGRAMA, *pers. comm.*). The estimated imports per sea area were calculated multiplying the total imports by the proportion per fishing area. Quantities presented in tonnes (t).

Country and fishing area	Production (t)	Proportion	Total imports (t)	Estimated imports (t)
**Morocco**				
Area 3 (FAO 34)	34,611	0.99		1,072
Area 1 (FAO 37)	454	0.01	1086	14
Total	35,065	1		1,086
**France**				
Area 2 (FAO 27)	2,636	0.52		1,725
Area 1 (FAO 37)	2,389	0.48	3289	1,564
Total	5,025	1.00		3,289
**Greece**				
Area 3 (FAO 37)	8,756		109	109
Total	8,756			109
**Italy**				
Area 1 (FAO 37)	29,664		3,872	3,872
Total	29,664			3,872
**Portugal**				
Area 2 (FAO 27)	387		359	359
Total	387			359
**United Kingdom**				
Area 2 (FAO 27)	10			6
Area 3 (FAO 34)	0		6	0
Total	10			6
**Croatia**				
Area 1 (FAO 37)	8,904		34	34
Total	8,904			34
**Norway**				
Area 2 (FAO 27)	NA*		1	1
Total	NA*			1
**Total Area 1 (FAO 37)**				5,593
**Total Area 2 (FAO 27)**				2,091
**Total Area 3 (FAO 34)**				1,072
**TOTAL**			8,756	8,756

* No reported captures of anchovies from Norway were available at FAO[1]. Therefore, it was assumed that the imported tonne of anchovy fished by Norway came from FAO 27.

Table S2. Spanish global production (production) of European anchovy[1], imports (import) (determined in Table S1) and exports (export) of fresh anchovy in tonnes (t) for 2013, estimated consumption and resulting proportions (Proportion) per fishing area (Area).

Area	Production (t)	Import (t)	Export (t)*	Estimated consumption (t)	Proportion
1	18,865	5,593	1,539	22,918.696	0.546
2	17,255	2,091	1,408	17,938.649	0.428
3	28	1,072	2	1,097.655	0.026
Total	36,148	8,756	2,949	41,955	1

* Total exports were provided by MAGRAMA (MAGRAMA, *pers. comm.*). The exports per fishing area were assumed in the same proportion to what was produced.

Table S3. The following questions and subsequent answers from questionnaire 1 were used to identify respondents at risk of anisakiasis caused by ingestion of raw and marinated anchovies at home.

**Code**	**Question**	**Response(s)**
18	Do you usually eat raw or undercooked fish?	Yes
18A	Where and how often?	Home (yearly, monthly, weekly)
18A2	What fish products do you use when preparing food containing raw or light cooked fish?	Fresh fish
18B	What fish?	Anchovy
18C1	What type of anchovy recipe or specialty?	In vinegar or lemon
		Pickled or marinated (eg. “escabeche”)
		Sushi or sashimi
		Ceviche
		Carpaccio

Table S4. Correction of questionnaire 1 by geographical location and population size. n: number of respondents; Nallmeal: number of anchovy meals consumed by respondents per year; Nuntreated: number of untreated anchovy meals consumed by respondents per year; Allergy: Number of respondents (out of those (n= 701) answering question 13S4 (Do you have allergy to *Anisakis*?)) with allergy to *Anisakis* spp.

Region	n[a]	Nallmeal	Nuntmeal	Allergy	Population[b]	Nuntmeal/ n	(Nuntmeal/n)*Population	(Nallmeal/n)*Population	Allergy /n	Allergic Population
Galicia	447	2,267	332	0	2,362,657	0.74	1,754,814	11,982,423	0	0
Central Spain	119	1,694	348	8	11,847,502	2.92	34,646,477	168,652,678	0	796,471
Mediterranean Sea	47	545	102	1	12,194,004	2.17	26,463,584	141,398,560	0.067	259,447
Cantabrian Sea	59	875	178	0	3,233,307	3.02	9,754,721	47,951,580	0.021	0
Andalusia	22	237	25	0	6,740,745	1.14	7,659,938	72,616,209	0	0
Canary Islands	16	147	0	0	1,738,480	0	0	15,972,289	0	0
Total	710[a]	5,765	985	9	38,116,695	NA	80,279,534	458,573,739	NA	1,055,918

Key variables that are used in the dose response and risk characterisation methods calculated from the corrected variables above.

Amealpy= Number of untreated anchovy meals eaten by Spanish person per year	Number of untreated meals eaten by respondents (n=716)	Propunt= Proportion of untreated anchovy meals consumed per person	Proportion allergic= Proportion of the Spanish population with allergy to *Anisakis* spp.	Allergic respondents= number of respondents with allergy to *Anisakis* spp.	Auntmeal= Number of untreated meals eaten by allergic respondents
80,279,534/38,116,695=	716*2.106=	80,279,534/458,573,739=	1,055,918/38,116,695=	701*0.028=	701*2.106=
**2.106**	**1,508**	**0.175**	**0.028**	**19**	**1,476**

[a] The region of some respondents (n=6) was unknown, so they were omitted from the analysis.
[b] Spanish population aged 18 and over (at 01_07_13) (Source: INE (Spanish Statistical Office) available from: http://www.ine.es/.)

Table S5. Calculation of the number of untreated anchovy meals eaten in 2013 utilised in risk characterization method 2 (Community scenario). Totalmass: kg of fresh anchovies consumed at home[2]; Musclemass: grams of muscle; Numberfillets: anchovy fillets; Numbermeals: number of anchovy meals; Nmunt: number of untreated anchovy meals.

RC method 2: Calculation of the anchovy meals consumed in Spain and the autonomous communities in 2013.

Variable	Totalmass (Kg)	Musclemass (g)	Numberfillets	Numbermeals	Nmunt
Formula		=Totalmass*1000 *0.51[a]	=Musclemass/4.25[b]	=Numberfillets/11[c]	=Numbermeals*(0.175)[d]
Total Spain	45,542,480	23,226,664,800	5,465,097,600	496,827,055	86,976,294
Andalucía	12,177,090	6,210,315,900	1,461,250,800	132,840,982	23,255,610
Aragón	1,044,430	532,659,300	125,331,600	11,393,782	1,994,636
Asturias	1,026,890	523,713,900	123,226,800	11,202,436	1,961,138
Baleares	511,310	260,768,100	61,357,200	5,577,927	976,492
Canarias	100,910	51,464,100	12,109,200	1,100,836	192,716
Cantabria	1,140,110	581,456,100	136,813,200	12,437,564	2,177,364
Castilla la Mancha	2,351,520	1,199,275,200	282,182,400	25,652,945	4,490,895
Castilla y León	2,499,090	1,274,535,900	299,890,800	27,262,800	4,772,722
Cataluña	6,213,760	3,169,017,600	745,651,200	67,786,473	11,866,939
Extremadura	871,200	444,312,000	104,544,000	9,504,000	1,663,804
Galicia	879,230	448,407,300	105,507,600	9,591,600	1,679,139
La Rioja	356,110	181,616,100	42,733,200	3,884,836	680,093

Comunidad de Madrid	7,017,070	3,578,705,700	842,048,400	76,549,855	13,401,087
Murcia	1,145,980	584,449,800	137,517,600	12,501,600	2,188,574
Navarra	574,600	293,046,000	68,952,000	6,268,364	1,097,362
País Vasco	3,650,260	1,861,632,600	438,031,200	39,821,018	6,971,208
Comunidad Valenciana	3,982,930	2,031,294,300	477,951,600	43,450,145	7,606,536

[a] Yield of a skinless fillet of European anchovy (i.e. 0.51)[3].
[b] Average mass of an anchovy fillet in grams (i.e. 4.25 g) (see subsection "Hospital studies" at "Hazard characterization" section).
[c] Average number of anchovy in vinegar fillets consumed per meal (11 fillets) (see subsection "RC scenarios" at "Risk characterization" section).
[d] Proportion of untreated anchovy meals calculated from corrected questionnaire 1 (see subsection "Consumption" at "Exposure assessment" section, and "Propunt" in Table S4).

"Anchovy meal size" sub-model.

This sub-model was implemented in Microsoft Excel™ using @RISK software (Palisade, UK) for estimating a probability distribution for the number of anchovy fillets consumed in a meal.

The second questionnaire (see supplementary materials) determined the number of fillets consumed at home per anchovy in vinegar meal and the frequency of consumption. A total of 35 respondents answered what was their minimum, most likely and maximum number of fillets consumed per meal (question 3) and their frequency of consumption (i.e. weekly, monthly, yearly) (question 4) (Table S6). It was assumed that a respondent ate 1, 12 or 52 meals per year when s/he answered annually, monthly or weekly anchovy consumption to question 4.

The number of fillets consumed per meal by a respondent was selected using a RiskTriang() distribution, in which the minimum, most likely and maximum number of "Fillets" consumed per meal were the parameters of the distribution (Table S7). The frequency of meals consumed per year by each respondent was stored in the variable "Fweek" (Table S6). A total of 229 anchovy in vinegar meals (Fwcum, Table S6) were eaten each year by the 35 respondents.

A random number (Rand, Table S7) was generated between 1 and 229 in order to select a particular anchovy in vinegar meal from a particular respondent (Sresp, Table S7) and number of fillets (Sfil, Table S7). Finally, the number of fillets consumed per meal was recorded (Mfillets, Table S7). This was repeated for 100,000 iterations and the output distribution is presented in Table S8. As a result, a meal can have from one to 39 anchovy fillets with an average of 11 anchovy fillets consumed per meal. These data were then used in the QRA model via the use of the RiskDiscrete() distribution (Nfillet, Table 1).

Table S6. Input data for sub-model "anchovy meal size". Responses from questionnaire 2 are listed below. Respondent: survey respondents. Fweek: number of anchovy meals eaten per year. Fwcum: cumulative of Fweek. Min., mlik. and max.: minimum, most likely and maximum number of anchovy fillets consumed by respondent per meal.

Respondent	Fweek	Fwcum	min	mlik	max
1	12	12	6	12	25
2	1	13	4	7	10
3	12	25	10	15	20
4	12	37	5	9	15
5	12	49	4	8	12
6	1	50	10	10	10
7	12	62	5	8	12
8	1	63	5	7	10
9	1	64	5	10	15
10	1	65	1	10	15
11	1	66	2	3	5
12	12	78	7	20	30
13	1	79	4	6	8
14	1	80	1	1	1
15	12	92	5	12	20
16	1	93	7	8	10
17	1	94	2	4	7
18	1	95	1	3	5
19	1	96	10	10	20
20	1	97	8	20	40
21	1	98	4	7	10
22	1	99	4	6	6
23	1	100	5	10	15
24	12	112	5	10	20
25	1	113	6	12	20
26	12	125	5	7	10
27	1	126	2	10	15

28	1	127	4	4	4
29	1	128	6	6	8
30	12	140	10	12	20
31	1	141	5	5	7
32	12	153	9	12	15
33	52	205	6	8	10
34	12	217	10	10	30
35	12	229	10	12	16

Table S7. Sub-model "anchovy meal size": Calculations to obtain the distribution that defines the number of anchovy in vinegar fillets consumed per meal and its frequency of consumption using information from questionnaire 2 provided in Table S6. Model variables: Fillets: selecting number of fillets per meal by respondent using the RiskTriang() function. Rand: random number generated between 1 and 35 (i.e. respondents). Sresp: selecting one respondent per iteration. Sfil: selecting the number of fillets consumed by the selected respondent. Mfillets: distribution of the number of meals consumed by respondents (the average number of anchovy fillets consumed by respondent can be also obtained ("Anfillet" in Table S10)).

Model variables				
Fillets	Rand	Sresp	Sfil	Mfillets
Round(RiskTriang(min1,mlik1,max1),0)	INT(RAND()*Fwcum35)	IF(Rand>-0.1,IF(Rand<(Fwcum+1),1,0),0)	Srep1*Fillets1	Sum(Sfil1:Sfil35)
Round(RiskTriang(min2,mlik2,max2),0)		IF(Rand>Fwcum1,IF(Rand<(Fwcum2+1),1,0),0)	Srep2*Fillets2	
…		…	…	
Round(RiskTriang(min35,mlik35,max35),0)		IF(Rand>Fwcum34,IF(Rand<(Fwcum35+1),1,0),0)	Srep35*Fillets35	

Table S8. Output distribution from Monte Carlo "anchovy meal size" sub-model (100,000 iterations) of the frequency of the number of fillets consumed per anchovy in vinegar meal.

Nfillets	Frequency	Fraction
1	462	0.00462
2	149	0.00149
3	558	0.00558
4	979	0.00979
5	1,445	0.01445
6	4,657	0.04657
7	11,593	0.11593
8	16,456	0.16456
9	11,275	0.11275
10	6,631	0.06631
11	6,786	0.06786
12	7,845	0.07845
13	6,864	0.06864
14	5,460	0.0546
15	4,265	0.04265
16	3,210	0.0321
17	2,688	0.02688
18	2,031	0.02031
19	1,555	0.01555
20	1,062	0.01062
21	890	0.0089
22	759	0.00759
23	603	0.00603
24	495	0.00495
25	401	0.00401
26	315	0.00315
27	256	0.00256
28	155	0.00155

29	77	0.00077
30	26	0.00026
31	6	0.00006
32	6	0.00006
33	14	0.00014
34	8	0.00008
35	5	0.00005
36	5	0.00005
37	3	0.00003
38	2	0.00002
39	3	0.00003

Table S9. Calculation of ID50 and R by method 1 (hospital studies) and 3 (questionnaire 1 (Qaire 1)). Variables; considered variables: Population, number of persons in the hospital influence area; Cases, number of anisakiasis cases in the studied population; Year, duration of the study in years; Casesyear, number of anisakiasis per year; Incidence, number of anisakiasis cases per 100,000 inhabitants/year; Amealpy, number of untreated anchovy meals consumed per person and year; Auntmeal, number of untreated anchovy meals consumed by study population; Pdisease, probability of disease caused by untreated anchovy meals; Dose1, Dose0.5, Dose0.1; average number of viable parasites in untreated anchovy meals when viability of *Anisakis* was 100%, 50% and 10%; R1, R0.5, R0.1: R value when *Anisakis* viability was 100%, 50% or 10%; ID50_1, ID50_0.5, ID50_0.1: ID50 value when *Anisakis* viability was 100%, 50% or 10%.

		Method 1 (Hospital studies)								Method 3 (Qaire 1)
		La Paz		Virgen de la Salud		Antequera		Carlos III		Qaire 1
Variables	Distribution, fixed number or calculation	Value	Reference	Value	Reference	Value	Reference	Value	Reference	Value
Population	Number	500,000	BIOHAZ (2010)	323,000	Repiso Ortega et al., 2003	110,000	Del Rey Moreno *pers. comm.*	490,000	González Muñoz *pers. comm.*	701
Cases	Number	96	Alonso Gómez et al., 2004	25	Repiso Ortega et al., 2003	52	Del Rey Moreno et al., 2008	30	González Muñoz *pers. comm.*	19[c]

Year	Number	1	Alonso Gómez et al., 2004	2	Repiso Ortega et al., 2003	4	Del Rey Moreno et al., 2008	1	González Muñoz *pers. comm.*	23.81
Casesyear	=Cases/Year	96	NA[a]	12.5	NA	13	NA	30	NA	0.4
Incidence	=(Cases/Year)*100000/Population	19.2	NA	3.87	Repiso Ortega et al., 2003	11.82	NA	6.12	NA	116.33
Amealpy	NA	2.106[b]	Table S4	2.106[b]	Table S4	2.106[b]	Table S4	2.106[b]	Table S4	NA
Auntmeal	=Amealpy*Population	1,053,076	NA	680,287	NA	231,677	NA	1,032,014	NA	1,476[d]
Pdisease	=Casesyear/Auntmeal	0.0000912	NA	0.0000184	NA	0.0000561	NA	0.0000291	NA	0.000552
Dose1	Average	0.66	QRA model	0.66	QRA model	0.66	QRA model	0.66	QRA model	0.66
R1	=-(1/Dose1)*LN(1-Pdisease)	0.0001381	NA	0.0000278	NA	0.0000850	NA	0.0000440	NA	0.0008371
ID50_1	-(LN(0.5)/R1)	5,018	NA	24,897	NA	8,153	NA	15,737	NA	828

Dose0.5	Average	0.45	QRA model	0.45	QRA model	0.45	QRA model	0.45	QRA model	0.45
R0.5	=-(1/Dose0.5)*LN(1-Pdisease)	0.000203	NA	0.0000408	NA	0.0001247	NA	0.0000646	NA	0.0012277
ID50_0.5	-(LN(0.5)/R0.5)	3,421	NA	16,975	NA	5,559	NA	10,730	NA	565
Dose0.1	Average	0.01	QRA model	0.01	QRA model	0.01	QRA model	0.01	QRA model	0.01
R0.1	=-(1/Dose0.1)*LN(1-Pdisease)	0.0091166	NA	0.0018375	NA	0.0056114	NA	0.0029070	NA	0.0552471
ID50_0.1	-(LN(0.5)/R0.1)	76	NA	377	NA	124	NA	238	NA	13

[a] NA - not applicable.
[b] Number of untreated anchovy meals eaten by Spanish person per year (see “Amealpy” in Table S4).
[c] Number of respondents with allergy to *Anisakis* spp. (see “Allergic respondents” in Table S4).
[d] Number of untreated meals eaten by allergic respondents (see “Auntmeal” in Table S4).

Table S10. Calculation of ID50 and R by method 2 (hospital studies). Variables; considered variables: Capita, consumption per capita of fresh anchovies at home (available at MAGRAMA (2016)); Cyear: kilograms of fresh anchovy consumed by studied population; Yield, yield of skinless anchovy fillets (FAO); Cfyear, kilograms of flesh consumed by population; Anfillet, average number of fillets consumed per meal by Spanish respondents; Amfillet, average weight of muscle of an anchovy fillet. Ammeal, average mass in kilograms of an anchovy meal; Nmealyear, number of anchovy meals consumed per year by population. Auntmeal: number of untreated anchovy meals consumed by population. Pdisease, R1, R0.5, R0.1, ID50_1, ID50_0.5, ID50_0.1 as described in Table S8.

Method 2 (Hospital studies)						
		La Paz	Virgen de la Salud	Antequera	Carlos III	Source of data
Variable	Distributional assumption	Value	Value	Value	Value	
Capita	Number	1.17	1.03	1.39	1.17	MAGRAMA[2]
Cyear	Capita*Population	585,000	332,690	152,900	573,300	
Yield	Number	0.51	0.51	0.51	0.51	
Cfyear	Cyear*Yield	298,350	169,672	77,979	292,383	
Anfillet	Average	11	11	11	11	
Amfillet	Average	0.00425	0.00425	0.00425	0.00425	
Ammeal	Anfillet*Amfillets	0.04675	0.04675	0.04675	0.04675	
Nmealyear	Cyear*Ammeal	12,513,369	7,116,364	1,668,000	12,263,102	

Propunt	NA[a]	0.175	0.175	0.175	0.175	Table S4
Auntmeal	=Propunt*Nmealyear	2,190,634	1,245,816	292,006	2,146,822	
Pdisease	Casesyear/Auntmeal	0.0000438	0.0000100	0.0000445	0.0000140	Casesyear in Table S9
R1	=-(1/Dose1)*LN(1- Pdisease)	0.0000664	0.0000152	0.0000675	0.0000212	Dose1 in Table S9
ID50_1	-(LN(0.5)/R1)	10,439	45,594	10,276	32,737	
R0.5	=-(1/Dose0.5)*LN(1- Pdisease)	0.0000974	0.0000223	0.000099	0.000031	Dose0.5 in Table S9
ID50_0.5	-(LN(0.5)/R0.5)	7,117	31,087	7,006	22,321	
R0.1	=-(1/Dose0.1)*LN(1- Pdisease)	0.0043824	0.0010034	0.004452	0.001397	Dose0.1 in Table S9
ID50_0.1	-(LN(0.5)/R0.1)	158	691	156	496	

[a] NA - not applicable.

Questionnaire 1 (Spanish version)

Seguridad alimentaria en productos de la pesca

Esta encuesta forma parte del proyecto 'PARASITE', que estudia la incidencia, los efectos y el control de gusanos nematodos en productos de la pesca. Se trata de un trabajo de gran importancia con el que se pretende aumentar la calidad y seguridad de los productos pesqueros. Sus respuestas serán estrictamente confidenciales y anónimas. Por favor, si usted es mayor de edad y vive en España, responda a las siguientes preguntas. Muchas gracias por su colaboración.

Hay 46 preguntas en esta encuesta

País de residencia

Por favor, confirme que vive en España. *

Por favor seleccione **sólo una** de las siguientes opciones:

- ○ España
- ○ Croacia
- ○ Dinamarca
- ○ Reino Unido

Preguntas generales

1) Sexo

Por favor seleccione **sólo una** de las siguientes opciones:

- Femenino
- Masculino

2) Edad

Por favor seleccione **sólo una** de las siguientes opciones:

- 18-29 años
- 30 -49 años
- 50-64 años
- 65 años o más

3) ¿Cuál es su país de origen?

Por favor seleccione **sólo una** de las siguientes opciones:

- España
- Croacia
- Dinamarca
- Reino Unido
- Otro ____________

Ej. Grecia

4) ¿En qué localidad vive actualmente? Indique también la provincia.

Por favor, escriba su respuesta aquí:

____

Ej.: Vigo (Pontevedra).

5) Estado civil

Por favor seleccione **sólo una** de las siguientes opciones:

- Casado/a o pareja de hecho
- Soltero

6) ¿Cuántos adultos viven en su hogar?

Sólo se pueden introducir números en este campo.

Por favor, escriba su respuesta aquí:

7) ¿Cuántos menores de 18 años viven en su hogar?

Sólo se pueden introducir números en este campo.

Por favor, escriba su respuesta aquí:

8) ¿Cuál es su nivel de estudios?

Por favor seleccione **sólo una** de las siguientes opciones:

- Sin escolarizar
- Educación Primaria
- Educación Secundaria
- Universitarios

9) Indique su situación laboral

Por favor seleccione **sólo una** de las siguientes opciones:

- Asalariado
- Trabajadores por cuenta propia
- Desempleado
- Jubilado
- Inactivo (ej. estudiantes, tareas domésticas, cuidado de los hijos)

10) Indique su principal sector o área de experiencia

Por favor seleccione **sólo una** de las siguientes opciones:

- Sector extractivo de la pesca (pescador, armador, etc.)
- Sector procesador de productos de la pesca
- Investigación y Desarrollo
- Actividades relacionadas con la salud humana
- Educación y formación (incluye a los estudiantes sin experiencia profesional)
- Industria relacionada con el suministro de energía o agua
- Extracción de minerales, combustibles; manufactura de metales, productos minerales y químicos
- Bienes de equipo, Industrias de ingeniería y automoción
- Otras industrias manufactureras
- Construcción
- Comercio al por menor y servicios de restauración
- Transporte y comunicaciones
- Actividades bancarias, finanzas, seguros, servicios empresariales y arrendamiento
- Otros

11) ¿Cuál es el ingreso bruto anual de su hogar?

Por favor seleccione **sólo una** de las siguientes opciones:

- 0 – 12000€
- 12001€ - 24000€
- 24001€ - 36000€
- 36001€ - 48000€
- 48001€ - 60000€
- 60001€ - 100000€
- Más de 100001€

Alergias

12) ¿Cómo calificaría su estado de salud?

Por favor seleccione **sólo una** de las siguientes opciones:

- Excelente
- Bueno
- Regular
- Malo

13) ¿Tiene alguna alergia?

Por favor seleccione **sólo una** de las siguientes opciones:

- Sí
- No

13. A) Si ha contestado afirmativamente a la anterior cuestión, por favor indique el o los tipos de alergias:

Sólo conteste esta pregunta si se cumplen las siguientes condiciones:
La respuesta fue 'Sí' en la pregunta '14 [A13]' (13) ¿Tiene alguna alergia?)

Por favor, marque las opciones que correspondan:

- Pescado
- Gambas
- Otros productos marinos
- Anisakis
- Otras alergias alimentarias
- Polvo
- Otras

Consumo de pescado

14) ¿Con qué frecuencia come pescado?

Por favor seleccione **sólo una** de las siguientes opciones:

- ○ Nunca
- ○ 1 vez al mes
- ○ 1 vez a la semana
- ○ 2 - 3 veces por semana
- ○ 4 veces o más por semana

15) ¿Cómo suele consumir el pescado?

Por favor, marque las opciones que correspondan:

- ☐ Fresco
- ☐ Congelado
- ☐ Productos surimi
- ☐ Otros (e.j. conservas)

16) ¿Qué especies de pescado suele consumir?

Por favor, indique la frecuencia de consumo de los pescados que consuma: Anual, Mensual o Semanal

Por favor, seleccione la respuesta apropiada para cada concepto:

	Anualmente	Mensualmente	Semanalmente
Merluza, pescadilla, lluç o legatza	○	○	○
Boquerón, anchoa, seitó o bokarte	○	○	○
Rape, peixe sapo, rap o zapo zuria (o sapo baltza)	○	○	○
Gallo, rapante, bruixa o itxas oillarra	○	○	○
Bacaladilla, lirio, maire o perlita	○	○	○
Jurel o chicharro, xurelo, sorell o txitxarro baltza	○	○	○
Bacalao, bacallao, bacallá o bakailaoa	○	○	○
Abadejo, abadexo o abadira	○	○	○
Caballa, xarda, verat o berdela	○	○	○
Sardina, sardiña o parrotxa	○	○	○
Atún	○	○	○
Salmón o trucha	○	○	○
Pez de San Pedro, San martiño, gall o muxumartin	○	○	○
Otros	○	○	○

17) ¿Dónde suele comer pescado?

Por favor seleccione **sólo una** de las siguientes opciones:

- ○ Casa
- ○ Otros (e.j. Restaurante)
- ○ Ambos lugares

Consumo de pescado crudo

18) ¿Suele comer pescado crudo o poco cocinado?

Por favor seleccione **sólo una** de las siguientes opciones:

○ Sí

○ No

Las recetas de pescado crudo o poco cocinado incluyen:

- Pescado poco hecho. (e.j. cocinado por fuera pero poco hecho por dentro).
- Pescado crudo macerado en limón o vinagre (ej. boquerones en vinagre).
- Pescado encurtido o marinado.
- Pescado ahumado (ej. salmón).
- Pescado en salmuera o salazón.
- Pescado Seco.
- Sushi o sashimi.
- Ceviche.

Conteste a estas preguntas si consume pescado crudo o ligeramente cocinado:

18. A) ¿Dónde y con qué frecuencia? Puede seleccionar una o dos opciones:

Por favor marque la casilla por frecuencia de consumo en los distintos lugares.

Sólo conteste esta pregunta si se cumplen las siguientes condiciones:
La respuesta fue 'Sí' en la pregunta '20 [A18]' (18) ¿Suele comer pescado crudo o poco cocinado?)

Por favor, seleccione la respuesta apropiada para cada concepto:

	Anualmente	Mensualmente	Semanalmente
Hogar	○	○	○
Otros sitios (ej. restaurantes)	○	○	○

18. A.2 ¿Qué tipo de pescado utiliza para comer crudo o poco cocinado?

Sólo conteste esta pregunta si se cumplen las siguientes condiciones:
La respuesta fue 'Semanalmente' *o* 'Mensualmente' *o* 'Anualmente' en la pregunta '21 [A18A]' (Conteste a estas preguntas si consume pescado crudo o ligeramente cocinado: 18. A) ¿Dónde y con qué frecuencia? Puede seleccionar una o dos opciones: Por favor marque la casilla por frecuencia de consumo en los distintos lugares. (Hogar))

Por favor, marque las opciones que correspondan:

- ☐ Pescado crudo ya preparado (ej. salmón ahumado envasado, sushi, etc.)
- ☐ Pescado fresco
- ☐ Pescado congelado

Si come pescado crudo o ligeramente cocinado:

18. B) ¿Con qué tipo de pescado?

Por favor, marque sólo el tipo de producto pesquero que consuma de modo crudo o ligeramente cocinado.

Sólo conteste esta pregunta si se cumplen las siguientes condiciones:
La respuesta fue 'Sí' en la pregunta '20 [A18]' (18) ¿Suele comer pescado crudo o poco cocinado?)

Por favor, marque las opciones que correspondan:

- ☐ Boquerón, anchoa, seitó o bocarte
- ☐ Sardina, sardiña o parrotxa
- ☐ Merluza, pescadilla, lluç o olegatza
- ☐ Caballa, xarda, verat o berdela
- ☐ Salmón o trucha
- ☐ Atún
- ☐ Rape, peixe sapo, rap, zapo zuria o sapo baltza
- ☐ Bacalao, bacallao, bacallá, bakailaoa
- ☐ Gallo, rapante, bruixa o itxas oillarra
- ☐ Calamares
- ☐
- ☐ Otro

Usted come **Boquerón, anchoa, seitó o bokarte** (crudo o ligeramente cocinado:

18. C1) ¿Qué tipo de recetas o especialidades consume? Es posible seleccionar distintas opciones.

Por favor marque la frecuencia de consumo en cada casilla sólo para aquellas recetas que consuma: anualmente, mensualmente, semanalmente

Sólo conteste esta pregunta si se cumplen las siguientes condiciones:
La respuesta fue en la pregunta '23 [A18B]' (Si come pescado crudo o ligeramente cocinado: 18. B) ¿Con qué tipo de pescado? Por favor, marque sólo el tipo de producto pesquero que consuma de modo crudo o ligeramente cocinado.)

Por favor, seleccione la respuesta apropiada para cada concepto:

	Anualmente	Mensualmente	Semanalmente
Poco cocinado (ej. cocinado por fuera, poco hecho por dentro)	○	○	○
En vinagre o al limón	○	○	○
Encurtido o marinado (ej. en escabeche)	○	○	○
Ahumado en frío	○	○	○
Sushi o sashimi	○	○	○
Ceviche	○	○	○
Seco	○	○	○
Salado	○	○	○
Carpaccio	○	○	○
Otros	○	○	○

Usted come **Sardina, sardiña o parrotxa** cruda o ligeramente cocinada:

18. C2) ¿Qué tipo de recetas o especialidades consume? Es posible seleccionar distintas opciones.

Por favor marque la frecuencia de consumo en cada casilla sólo para aquellas recetas que consuma: anualmente, mensualmente, semanalmente

Sólo conteste esta pregunta si se cumplen las siguientes condiciones:
La respuesta fue en la pregunta '23 [A18B]' (Si come pescado crudo o ligeramente cocinado: 18. B) ¿Con qué tipo de pescado? Por favor, marque sólo el tipo de producto pesquero que consuma de modo crudo o ligeramente cocinado.)

Por favor, seleccione la respuesta apropiada para cada concepto:

	Anualmente	Mensualmente	Semanalmente
Poco cocinada (ej. cocinada por fuera, poco hecho por dentro)	○	○	○
Al limón o con vinagre	○	○	○
Encurtida o marinada (ej. en escabeche)	○	○	○
Ahumada en frío	○	○	○
Sushi o sashimi	○	○	○
Ceviche	○	○	○
Seca	○	○	○
Salada	○	○	○
Carpaccio	○	○	○
Otra	○	○	○

Usted come **Merluza, pescadilla, lluç o olegatza** cruda o ligeramente cocinada:

18. C3) ¿Qué tipo de recetas o especialidades consume? Es posible seleccionar distintas opciones.

Por favor marque la frecuencia de consumo en cada casilla sólo para aquellas recetas que consuma: anualmente, mensualmente, semanalmente

Sólo conteste esta pregunta si se cumplen las siguientes condiciones:
La respuesta fue en la pregunta '23 [A18B]' (Si come pescado crudo o ligeramente cocinado: 18. B) ¿Con qué tipo de pescado? Por favor, marque sólo el tipo de producto pesquero que consuma de modo crudo o ligeramente cocinado.)

Por favor, seleccione la respuesta apropiada para cada concepto:

	Anualmente	Mensualmente	Semanalmente
Poco cocinada (ej. cocinada por fuera, poco hecha por dentro)	○	○	○
Al limón o con vinagre	○	○	○
Encurtida o marinada	○	○	○
Ahumada en frío	○	○	○
Sushi o sashimi	○	○	○
Ceviche	○	○	○
Seca	○	○	○
Salada	○	○	○
Carpaccio	○	○	○
Otros	○	○	○

Usted come Caballa, xarda, verat o berdela cruda o ligeramente cocinada:

18. C4) ¿Qué tipo de recetas o especialidades consume? Es posible seleccionar distintas opciones.

Por favor marque la frecuencia de consumo en cada casilla sólo para aquellas recetas que consuma: anualmente, mensualmente, semanalmente

Sólo conteste esta pregunta si se cumplen las siguientes condiciones:
La respuesta fue en la pregunta '23 [A18B]' (Si come pescado crudo o ligeramente cocinado: 18. B) ¿Con qué tipo de pescado? Por favor, marque sólo el tipo de producto pesquero que consuma de modo crudo o ligeramente cocinado.)

Por favor, seleccione la respuesta apropiada para cada concepto:

	Anualmente	Mensualmente	Semanalmente
Poco cocinada (ej. cocinada por fuera, poco hecha por dentro)	○	○	○
Al limón o con vinagre	○	○	○
Encurtida o marinada (ej. en escabeche)	○	○	○
Ahumada en frío	○	○	○
Sushi o sashimi	○	○	○
Ceviche	○	○	○
Seca	○	○	○
Salada	○	○	○
Carpaccio	○	○	○
Otros	○	○	○

Usted come Salmón o trucha crudo o ligeramente cocinado:

18. C5) ¿Qué tipo de recetas o especialidades consume? Es posible seleccionar distintas opciones.

Por favor marque la frecuencia de consumo en cada casilla sólo para aquellas recetas que consuma: anualmente, mensualmente, semanalmente

Sólo conteste esta pregunta si se cumplen las siguientes condiciones:
La respuesta fue en la pregunta '23 [A18B]' (Si come pescado crudo o ligeramente cocinado: 18. B) ¿Con qué tipo de pescado? Por favor, marque sólo el tipo de producto pesquero que consuma de modo crudo o ligeramente cocinado.)

Por favor, seleccione la respuesta apropiada para cada concepto:

	Anualmente	Mensualmente	Semanalmente
Poco cocinado (ej. cocinado por fuera, poco hecho por dentro)	○	○	○
Al limón o con vinagre	○	○	○
Encurtido o marinado	○	○	○
Ahumado en frío (ej. salmón o trucha)	○	○	○
Sushi o sashimi	○	○	○
Ceviche	○	○	○
Seco	○	○	○
Salado	○	○	○
Carpaccio	○	○	○
Otros	○	○	○

Usted come Atún crudo o ligeramente cocinado:

18. C6) ¿Qué tipo de recetas o especialidades consume? Es posible seleccionar distintas opciones.

Por favor marque la frecuencia de consumo en cada casilla sólo para aquellas recetas que consuma: anualmente, mensualmente, semanalmente

Sólo conteste esta pregunta si se cumplen las siguientes condiciones:
La respuesta fue en la pregunta '23 [A18B]' (Si come pescado crudo o ligeramente cocinado: 18. B) ¿Con qué tipo de pescado? Por favor, marque sólo el tipo de producto pesquero que consuma de modo crudo o ligeramente cocinado.)

Por favor, seleccione la respuesta apropiada para cada concepto:

	Anualmente	Mensualmente	Semanalmente
Poco cocinado (ej. cocinado por fuera, poco hecho por dentro)	○	○	○
Atún al limón o con vinagre	○	○	○
Encurtido o marinado (ej. en escabeche)	○	○	○
Ahumado en frío	○	○	○
Sushi o sashimi	○	○	○
Ceviche	○	○	○
Seco	○	○	○
Salado	○	○	○
Carpaccio	○	○	○
Otro	○	○	○

Usted come **Rape, peixe sapo, rap o zapo zuria (o sapo baltza)** crudo o ligeramente cocinado:

18. C7) ¿Qué tipo de recetas o especialidades consume? Es posible seleccionar distintas opciones.

Por favor marque la frecuencia de consumo en cada casilla sólo para aquellas recetas que consuma: anualmente, mensualmente, semanalmente

Sólo conteste esta pregunta si se cumplen las siguientes condiciones:
La respuesta fue en la pregunta '23 [A18B]' (Si come pescado crudo o ligeramente cocinado: 18. B) ¿Con qué tipo de pescado? Por favor, marque sólo el tipo de producto pesquero que consuma de modo crudo o ligeramente cocinado.)

Por favor, seleccione la respuesta apropiada para cada concepto:

	Anualmente	Mensualmente	Semanalmente
Poco cocinado (ej. cocinado por fuera, poco hecho por dentro)	○	○	○
Al limón o con vinagre	○	○	○
Encurtido o marinado	○	○	○
Ahumado en frío	○	○	○
Sushi o sashimi	○	○	○
Ceviche	○	○	○
Seco	○	○	○
Salado	○	○	○
Carpaccio	○	○	○
Otro	○	○	○

Usted come **Bacalao, bacallao, bacallá o bakailaoa** crudo o ligeramente cocinado:

18. C8) ¿Qué tipo de recetas o especialidades consume? Es posible seleccionar distintas opciones.

Por favor marque la frecuencia de consumo en cada casilla sólo para aquellas recetas que consuma: anualmente, mensualmente, semanalmente

Sólo conteste esta pregunta si se cumplen las siguientes condiciones:
La respuesta fue en la pregunta '23 [A18B]' (Si come pescado crudo o ligeramente cocinado: 18. B) ¿Con qué tipo de pescado? Por favor, marque sólo el tipo de producto pesquero que consuma de modo crudo o ligeramente cocinado.)

Por favor, seleccione la respuesta apropiada para cada concepto:

	Anualmente	Mensualmente	Semanalmente
Poco cocinado (ej. cocinado por fuera, poco hecho por dentro)	○	○	○
Al limón o con vinagre	○	○	○
Encurtido o marinado (ej. en escabeche)	○	○	○
Ahumado en frío	○	○	○
Sushi o sashimi	○	○	○
Ceviche	○	○	○
Seco	○	○	○
Salado	○	○	○
Carpaccio	○	○	○
Otro	○	○	○

Usted come **Gallo, rapante, bruixa o itxas oillarra** crudo o ligeramente cocinado:

18. C9) ¿Qué tipo de recetas o especialidades consume? Es posible seleccionar distintas opciones.

Por favor marque la frecuencia de consumo en cada casilla sólo para aquellas recetas que consuma: anualmente, mensualmente, semanalmente

Sólo conteste esta pregunta si se cumplen las siguientes condiciones:
La respuesta fue en la pregunta '23 [A18B]' (Si come pescado crudo o ligeramente cocinado: 18. B) ¿Con qué tipo de pescado? Por favor, marque sólo el tipo de producto pesquero que consuma de modo crudo o ligeramente cocinado.)

Por favor, seleccione la respuesta apropiada para cada concepto:

	Anualmente	Mensualmente	Semanalmente
Poco cocinado (ej. cocinado por fuera, poco hecho por dentro)	○	○	○
Al limón o con vinagre	○	○	○
Encurtido o marinado	○	○	○
Ahumado en frío	○	○	○
Sushi o sashimi	○	○	○
Ceviche	○	○	○
Seco	○	○	○
Salado	○	○	○
Carpaccio	○	○	○
Otro	○	○	○

Usted come Calamares crudos o ligeramente cocinados:

18. C10) ¿Qué tipo de recetas o especialidades consume? Es posible seleccionar distintas opciones.

Por favor marque la frecuencia de consumo en cada casilla sólo para aquellas recetas que consuma: anualmente, mensualmente, semanalmente

Sólo conteste esta pregunta si se cumplen las siguientes condiciones:
La respuesta fue en la pregunta '23 [A18B]' (Si come pescado crudo o ligeramente cocinado: 18. B) ¿Con qué tipo de pescado? Por favor, marque sólo el tipo de producto pesquero que consuma de modo crudo o ligeramente cocinado.)

Por favor, seleccione la respuesta apropiada para cada concepto:

	Anualmente	Mensualmente	Semanalmente
Poco cocinado (ej. cocinado por fuera, poco hecho por dentro)	○	○	○
Al limón o en vinagre	○	○	○
Encurtido o marinado	○	○	○
Ahumado en frío	○	○	○
Sushi o sashimi	○	○	○
Ceviche	○	○	○
Seco	○	○	○
Salado	○	○	○
Carpaccio	○	○	○
Otro	○	○	○

Sólo conteste esta pregunta si se cumplen las siguientes condiciones:
La respuesta fue en la pregunta '23 [A18B]' (Si come pescado crudo o ligeramente cocinado: 18. B) ¿Con qué tipo de pescado? Por favor, marque sólo el tipo de producto pesquero que consuma de modo crudo o ligeramente cocinado.)

Por favor, seleccione la respuesta apropiada para cada concepto:

○	○	○
○	○	○
○	○	○
○	○	○
○	○	○
○	○	○
○	○	○
○	○	○
○	○	○
○	○	○

Usted come Otros crudos o ligeramente cocinados:

18. C12) Por favor escriba que tipo de pescado consume, y la frecuencia con la que lo hace (anual, mensual, semanal) en la casilla correspondiente.

Sólo conteste esta pregunta si se cumplen las siguientes condiciones:
La respuesta fue en la pregunta '23 [A18B]' (Si come pescado crudo o ligeramente cocinado: 18. B) ¿Con qué tipo de pescado? Por favor, marque sólo el tipo de producto pesquero que consuma de modo crudo o ligeramente cocinado.)

Por favor, seleccione todas las opciones que correspondan y escriba un comentario:

- ☐ Pescado poco cocinado (ej. cocinado por fuera, poco hecho por dentro)
- ☐ Pescado al limón o con vinagre (ej. Boquerones)
- ☐ Encurtido o marinado (ej. en escabeche)
- ☐ Ahumado en frío (ej.salmón o trucha)
- ☐ Sushi o sashimi
- ☐ Ceviche
- ☐ Pescado seco
- ☐ Pescado salado
- ☐ Carpaccio
- ☐ Otro

Conocimiento sobre Anisakis

19) ¿Ha oído hablar del *Anisakis* o del “gusano del bacalao”o de “gusanos del pescado”?

Por favor seleccione **sólo una** de las siguientes opciones:

- Sí
- No

20) ¿Conoce algún método de prevención contra el *Anisakis* o los” gusanos del pescado”?

Por favor seleccione **sólo una** de las siguientes opciones:

- Sí
- No

20. A) Si ha contestado que sí a la anterior cuestión: Por favor, marque la casilla con los métodos que considera recomendados para prevenir las consecuencias de la presencia de los *Anisakis* o gusanos del pescado.

Sólo conteste esta pregunta si se cumplen las siguientes condiciones:
La respuesta fue 'Sí' en la pregunta '37 [A20]' (20) ¿Conoce algún método de prevención contra el Anisakis o los” gusanos del pescado”?)

Por favor, marque las opciones que correspondan:

- [ ] Comer pescado crudo o ligeramente cocinado
- [ ] Quitar pronto las tripas al pescado
- [ ] Cocinar el pescado
- [ ] Marinar o ahumar el pescado
- [ ] Congelar el pescado

21) ¿Alguna vez ha dejado de comprar o consumir pescado por la presencia de *Anisakis*?

Por favor seleccione **sólo una** de las siguientes opciones:

- Sí
- No

21. A) Si ha contestado que sí a la anterior cuestión, ¿qué especie ha sido?

Sólo conteste esta pregunta si se cumplen las siguientes condiciones:
La respuesta fue 'Sí' en la pregunta '39 [A21]' (21) ¿Alguna vez ha dejado de comprar o consumir pescado por la presencia de Anisakis?)

Por favor, marque las opciones que correspondan:

- ☐ Merluza, pescadilla, lluç o olegatza
- ☐ Bacalao, bacallao,, bacallá o bakailaoa
- ☐ Palometa o japuta, castañeta, castanyola o papardoa
- ☐ Sardina, sardiña o parrotxa
- ☐ Salmón o trucha
- ☐ Rape, peixe sapo, rap o zapo zuria (o sapo baltza)
- ☐ Atún
- ☐ Bacaladilla, lirio, maire o perlita
- ☐ Gallo, rapante, bruixa o itxas oillarra
- ☐ Caballa, xarda, verat o berdela
- ☐ Boquerón, anchoa, seitó o bokarte
- ☐ Otro: ____________

Anisakis, anisakiasis y alergia al Anisakis

Por favor, lea detenidamente la siguiente información sobre el *Anisakis* o el "gusano del pescado" antes de responder a la cuestión.

***Anisakis* o "gusano del pescado" es un gusano presente de manera natural en muchos pescados marinos.**

- **Cómo puede contraerse: este gusano puede provocar diversas enfermedades en humanos que consuman pescado crudo o ligeramente cocinado sin métodos preventivos.**
- **Gravedad: La enfermedad llamada anisakiasis puede cursar con gravedad leve a severa, incluyendo síntomas asociados a reacciones alérgicas.**
- **Probabilidad: Este parásito se considera un problema de salud emergente a nivel mundial, especialmente en Japón, España e Italia donde el pescado crudo, ligeramente cocinado o marinado, es parte de la dieta habitual (sushi, sashimi, boquerones en vinagre, ceviche, etc.). Además, en lo concerniente a las alergias alimentarias, datos publicados sugieren que el *Anisakis* es la mayor causa subyacente de reacciones alérgicas en España*.**
- **Prevención: Hasta la fecha, sabemos que para matar al gusano *Anisakis* hay que congelar o cocinar correctamente el pescado. Sin embargo, con frecuencia las personas que padezcan alergia a este parásito continuarán mostrando síntomas de alergia después de haber consumido pescado infectado, aunque éste haya sido congelado, cocinado o incluso procesado adecuadamente.**

Nota: Consulte la información disponible en el Panel de Riesgos Biológicos en página de la EFSA (European Food Safety Authority) si precisa más información sobre el *Anisakis* y enfermedades asociadas.

*** Fuente: Ver el enlace. ***

Por favor seleccione **sólo una** de las siguientes opciones:

- ○ Sí
- ○ No

Por favor, confirme la lectura de la información previa.

Disponibilidad de pago

23) Por favor, tenga en cuenta el siguiente escenario o situación y responda a la cuestión:

Suponga que haya sido descubierta una tecnología o un tratamiento que garantice que el *Anisakis* y sus características alergénicas puedan ser completamente eliminadas del pescado, siendo un tratamiento 100% efectivo que además no altere la calidad del pescado.

En ese caso, si su pescado favorito (ej. bacalao) costase 6€ por kilogramo en el supermercado:

¿Cuál sería el precio máximo que estaría dispuesto y podría pagar por el mismo producto libre de *Anisakis* y de sus características alergénicas?

Por favor seleccione **sólo una** de las siguientes opciones:

- ○ No compraría ese producto
- ○ 0% extra: 6 €/Kg (Lo compraría al precio establecido de 6€)
- ○ 10% extra : 6.60 €/Kg
- ○ 20% extra: 7.20 €/Kg
- ○ 30% extra: 7.80 €/Kg
- ○ 40% extra: 8.40 €/Kg
- ○ 50% extra: 9 €/Kg
- ○ 60% extra: 9.60 €/Kg
- ○ 70% extra: 10.20 €/Kg
- ○ 80% extra: 10.80 €/Kg
- ○ 90% extra: 11.40 €/Kg
- ○ 100% extra: 12 €/Kg o más

23. A) ¿Cuál es la razón por la que no está dispuesto a pagar más por ese tratamiento en el pescado?

Sólo conteste esta pregunta si se cumplen las siguientes condiciones:
La respuesta fue 'No compraría ese producto' *o* '0% extra: 6 €/Kg (Lo compraría al precio establecido de 6€)' en la pregunta '42 [A23]' (23) Por favor, tenga en cuenta el siguiente escenario o situación y responda a la cuestión: Suponga que haya sido descubierta una tecnología o un tratamiento que garantice que el Anisakis y sus características alergénicas puedan ser completamente eliminadas del pescado, siendo un tratamiento 100% efectivo que además no altere la calidad del pescado. En ese caso, si su pescado favorito (ej. bacalao) costase 6€ por kilogramo en el supermercado: ¿Cuál sería el precio máximo que estaría dispuesto y podría pagar por el mismo producto libre de Anisakis y de sus características alergénicas?)

Por favor, marque las opciones que correspondan:

- ☐ El riesgo es demasiado pequeño para justificar la diferencia de precio
- ☐ El escenario propuesto no es realista
- ☐ No se debería pagar ningún coste extra por una alimentación segura
- ☐ Otro: __________

24) ¿Evitaría comprar o comer pescado por la presencia de *Anisakis* o gusanos?

Por favor seleccione **sólo una** de las siguientes opciones:

- ○ Sí, siempre
- ○ Sí, pero sólo si existiese una alta probabilidad de que el pescado contenga gusanos
- ○ No, porque cocinar o congelar el pescado mataría a cualquier gusano

24. A) ¿Por qué evitaría comprar o comer pescado con *Anisakis*?

Sólo conteste esta pregunta si se cumplen las siguientes condiciones:
La respuesta fue 'Sí, siempre' *o* 'Sí, pero sólo si existiese una alta probabilidad de que el pescado contenga gusanos ' en la pregunta '44 [A24]' (24) ¿Evitaría comprar o comer pescado por la presencia de Anisakis o gusanos?)

Por favor seleccione **sólo una** de las siguientes opciones:

- ○ Riesgo de salud
- ○ Poco apetecible
- ○ Ambos motivos
- ○ Otro __________

Percepción del riesgo

25) Aunque por supuesto nadie puede asegurar qué sucederá en el futuro, nos gustaría saber qué probabilidades considera que tiene usted para desarrollar Anisakiasis o/y una alergia al *Anisakis*.

Por favor, use una escala de 0-4 donde 0 significa ninguna probabilidad y 4 absoluta seguridad de desarrollarla.

Por favor seleccione **sólo una** de las siguientes opciones:

- 0 (ninguna probabilidad)
- 1 (escasa probabilidad)
- 2 (probabilidad moderada)
- 3 (alta probabilidad)
- 4 (absoluta seguridad)

¡Muchas gracias por su colaboración!

Enviar su encuesta.
Gracias por completar esta encuesta.

Questionnaire 2. Consumption of anchovies in vinegar at Spanish homes.

1) What is your gender?
Please choose only one of the following:
- Female
- Male

2) What is your age?
Please choose only one of the following:
- 18-29 years old
- 30-49 years old
- 50-64 years old
- 65 years and over

3) How many anchovies in vinegar do you consume at home?
Please write your answer here:
- Minimum number of fillets per meal:__
- Most likely number of fillets per meal:__
- Maximum number of fillets per meal:__

4) Please, how often do you consume anchovies in vinegar at home?
Please choose only one of the following:
- Weekly
- Monthly
- Yearly

Questionnaire 1 (English version)

Food safety in fishery products

This questionnaire is part of the PARASITE Project, funded by the European Commission, which aims to provide new scientific evidence and technological developments to detect, monitor, and mitigate impacts of parasites occurring in European and imported fishery products, thus addressing concerns identified by European Food Safety Authority (EFSA). The University of Aberdeen (Scotland) is coordinating this survey in collaboration with several European research institutions and universities "Instituto de Investigaciones Marinas" and "Centro Tecnológico del Mar" (Spain), Institute of Oceanography and Fisheries (Croatia) and University of Copenhagen (Denmark)). The results of the study will be published in the final report of the project and in scientific papers.

Your replies will be treated as completely *confidential* and the questionnaire responses are *anonymous*. Please proceed only if you are currently living Scotland and you are aged 18 or over. Completing the questionnaire will be taken to imply that you provide the researchers with your informed consent to use your responses anonymously for the planned research. If you require any further information or have any comments or complaints, etc. please contact one of the project leads at the University of Aberdeen; Graham Pierce g.j.pierce@abdn.ac.uk or Ioannis Theodossiou theod@andn.ac.uk or Norval Strachan n.strachan@abdn.ac.uk.

For further information about this project please visit the project website http://parasite-project.eu/.

There are 46 questions in this survey

Country of residence

Please, confirm your country of residence. *

Please choose **only one** of the following:

- Spain
- Croatia
- Denmark
- Scotland

General questions

1) What is your gender?

Please choose **only one** of the following:

- Female
- Male

2) What is your age?

Please choose **only one** of the following:

- 18-29 years old
- 30 -49 years old
- 50-64 years old
- 65 years and over

3) In which country did you grow up?

Please choose **only one** of the following:

- Spain
- Croatia
- Denmark
- Scotland
- Other

E.g. Greece

4) In which city/town do you currently live? Including county councils.

Please write your answer here:

E.g.: Inverurie (Aberdeenshire).

5) Are You?

Please choose **only one** of the following:

- Married or cohabiting
- Single

6) How many adults live in your household?

Only numbers may be entered in this field.

Please write your answer here:

7) How many children (<18 years) live in your household?

Only numbers may be entered in this field.

Please write your answer here:

8) What is the highest educational qualification you have obtained?

Please choose **only one** of the following:

- No education
- Primary education
- Secondary education
- College education or university degree

9) Please indicate your employment status:

Please choose **only one** of the following:

- Employed
- Self-employed
- Unemployed
- Retired
- Labour market inactive (e.g. students, domestic activity, child rearing)

10) Please indicate below your own main occupation or expertise.

Please choose **only one** of the following:

- Extractive fishing sector (fisherman, owner, etc.)
- Processing sector of fishery products
- Science and Development
- Human Health activities
- Education and Training (including students without work experience)
- Energy & water supplies
- Extraction of minerals & ores other than fuels; manufacture of metals, mineral products & chemicals
- Metal goods, engineering & vehicles industries
- Other manufacturing industries
- Construction
- Distribution, hotels & catering, restaurants & pubs
- Transport & communication
- Banking, finance, insurance, business services & leasing
- Other

11) What is your total annual household income before tax?

Please choose **only one** of the following:

- 0 - £12000
- £12001 - £24000
- £24001 - £36000
- £36001 - £48000
- £48001 - £60000
- £60001 - £100000
- £100001 and above

Allergies

12) How would you judge your health?

Please choose **only one** of the following:

- Excellent
- Good
- Fair
- Poor

13) Have you got an allergy?

Please choose **only one** of the following:

- Yes
- No

13. A) You said yes to the previous question, please indicate which allergies you have:

Only answer this question if the following conditions are met:
Answer was 'Yes' at question '14 [A13]' (13) Have you got an allergy?)

Please choose **all** that apply:

- Fish
- Shrimp
- Other seafood
- Anisakis
- Other food allergies
- House dust mites
- Other

Fish consumption habits

14) How many times do you eat fish?

Please choose **only one** of the following:

- ○ Never
- ○ 1 per month
- ○ 1 per week
- ○ 2 - 3 times per week
- ○ 4 times or more per week

15) Which kind of fish do you usually eat?

Please choose **all** that apply:

- ☐ Fresh
- ☐ Frozen
- ☐ Surimi products
- ☐ Other (e.g. canned)

16) Which fish species do you usually eat?

Please tick only the following fish in their corresponding box if you eat them: Yearly, Monthly or Weekly.

Please choose the appropriate response for each item:

	Yearly	Monthly	Weekly
Hake	○	○	○
Anchovy	○	○	○
Herring	○	○	○
Flatfish (Plaice, sole, etc.)	○	○	○
Blue whiting	○	○	○
Haddock	○	○	○
Cod	○	○	○
Pollock	○	○	○
Mackerel	○	○	○
Sardine	○	○	○
Tuna	○	○	○
Salmon or trout	○	○	○
Whiting	○	○	○
Other	○	○	○

17) Where do you usually eat fish?

Please choose **only one** of the following:

- ○ Home
- ○ Other (e.g. restaurant)
- ○ Both

Consumption of raw fish

18) Do you eat any of the following raw or lightly cooked fish?

Please choose **only one** of the following:

- Yes
- No

Raw or lightly cooked fish include dishes like:

- Undercooked fish. (e.g. cooked on outside and raw in middle).
- Fish with lemon or vinegar.
- Pickled/marinated fish (e.g. soused herring or rollmops).
- Cold-smoked fish (e.g. salmon) or gravlax.
- Brined or salted fish.
- Dried fish.
- Sushi or sashimi.
- Ceviche.

If you eat raw or lightly cooked fish:

18. A) Where and how often? One or two choices are possible:

<u>Please tick</u> the corresponding box in terms of how often you eat them in the different places.

Only answer this question if the following conditions are met:
Answer was 'Yes' at question '20 [A18]' (18) Do you eat any of the following raw or lightly cooked fish?)

Please choose the appropriate response for each item:

	Yearly	Monthly	Weekly
Home	○	○	○
Other (e.g. restaurant)	○	○	○

If you eat raw or lightly cooked fish at home:

18. A.2 Do you use?

Only answer this question if the following conditions are met:
Answer was 'Weekly' *or* 'Monthly' *or* 'Yearly' at question '21 [A18A]' (If you eat raw or lightly cooked fish: 18. A) Where and how often? One or two choices are possible: Please tick the corresponding box in terms of how often you eat them in the different places. (Home))

Please choose **all** that apply:

- ☐ Already prepared raw fish (e.g. packed smoked salmon, rollmops, sushi, etc.)
- ☐ Fresh fish
- ☐ Previously frozen fish

If you eat raw or lightly cooked fish:

18. B) Which fish?

Please tick only the following fish in their corresponding box if you eat them.

Only answer this question if the following conditions are met:
Answer was 'Yes' at question '20 [A18]' (18) Do you eat any of the following raw or lightly cooked fish?)

Please choose **all** that apply:

- ☐ Anchovy
- ☐ Sardine
- ☐ Herring
- ☐ Mackerel
- ☐ Salmon or trout
- ☐ Tuna
- ☐ Haddock
- ☐ Cod
- ☐ Flatfish (e.g. plaice, sole, etc.)
- ☐ Squid
- ☐
- ☐ Other

You eat raw or lightly cooked Anchovy:

18. C1) Which type of recipes or specialties? Multiple choices are possible.

Please tick only the following fish in their corresponding box if you eat them: yearly, monthly; weekly.

Only answer this question if the following conditions are met:
Answer was at question '23 [A18B]' (If you eat raw or lightly cooked fish: 18. B) Which fish? Please tick only the following fish in their corresponding box if you eat them.)

Please choose the appropriate response for each item:

	Yearly	Monthly	Weekly
Undercooked anchovy (e.g. raw in middle)	○	○	○
Anchovies with vinegar or lemon	○	○	○
Pickled or other marinating process	○	○	○
Cold-smoked	○	○	○
Sushi or sashimi	○	○	○
Ceviche	○	○	○
Dried	○	○	○
Brined or salted	○	○	○
Carpaccio	○	○	○
Others	○	○	○

You eat raw or lightly cooked Sardine:

18. C2) Which type of recipes or specialties? Multiple choices are possible.

Please tick only the following fish in their corresponding box if you eat them: yearly, monthly; weekly.

Only answer this question if the following conditions are met:
Answer was at question '23 [A18B]' (If you eat raw or lightly cooked fish: 18. B) Which fish? Please tick only the following fish in their corresponding box if you eat them.)

Please choose the appropriate response for each item:

	Yearly	Monthly	Weekly
Undercooked sardine (e.g. raw in middle)	○	○	○
Sardine with lemon or vinegar	○	○	○
Pickled or other marinating process	○	○	○
Cold-smoked	○	○	○
Sushi or sashimi	○	○	○
Ceviche	○	○	○
Dried	○	○	○
Brined or salted	○	○	○
Carpaccio	○	○	○
Other	○	○	○

You eat raw or lightly cooked Herring:

18. C3) Which type of recipes or specialties? Multiple choices are possible.

Please tick only the following fish in their corresponding box if you eat them: yearly, monthly; weekly.

Only answer this question if the following conditions are met:
Answer was at question '23 [A18B]' (If you eat raw or lightly cooked fish: 18. B) Which fish? Please tick only the following fish in their corresponding box if you eat them.)

Please choose the appropriate response for each item:

	Yearly	Monthly	Weekly
Undercooked herring (e.g. raw in middle)	○	○	○
Herring with lemon or vinegar	○	○	○
Pickled or other marinating process (e.g. soused herring and rollmops)	○	○	○
Cold-smoked (e.g. kippers)	○	○	○
Sushi or sashimi	○	○	○
Ceviche	○	○	○
Dried	○	○	○
Brined or salted	○	○	○
Carpaccio	○	○	○
Other	○	○	○

You eat raw or lightly cooked Mackerel:

18. C4) Which type of recipes or specialties? Multiple choices are possible.

Please tick only the following fish in their corresponding box if you eat them: yearly, monthly; weekly.

Only answer this question if the following conditions are met:
Answer was at question '23 [A18B]' (If you eat raw or lightly cooked fish: 18. B) Which fish? Please tick only the following fish in their corresponding box if you eat them.)

Please choose the appropriate response for each item:

	Yearly	Monthly	Weekly
Undercooked mackerel (e.g. raw in middle)	○	○	○
Mackerel with lemon or vinegar	○	○	○
Pickled or other marinating process	○	○	○
Cold-smocked	○	○	○
Sushi or sashimi	○	○	○
Ceviche	○	○	○
Dried	○	○	○
Brined or salted	○	○	○
Carpaccio	○	○	○
Other	○	○	○

You eat raw or lightly cooked Salmon or trout:

18. C5) Which type of recipes or specialties? Multiple choices are possible.

Please tick only the following fish in their corresponding box if you eat them: yearly, monthly; weekly.

Only answer this question if the following conditions are met:
Answer was at question '23 [A18B]' (If you eat raw or lightly cooked fish: 18. B) Which fish? Please tick only the following fish in their corresponding box if you eat them.)

Please choose the appropriate response for each item:

	Yearly	Monthly	Weekly
Undercooked salmon (e.g. raw in middle)	○	○	○
Salmon with lemon or vinegar	○	○	○
Pickled or other marinating process	○	○	○
Cold-smoked (e.g. salmon or trout)	○	○	○
Sushi or sashimi	○	○	○
Ceviche	○	○	○
Dried	○	○	○
Brined or salted (e.g. gravlax)	○	○	○
Carpaccio	○	○	○
Other	○	○	○

You eat raw or lightly cooked Tuna:

18. C6) Which type of recipes or specialties? Multiple choices are possible.

Please tick only the following fish in their corresponding box if you eat them: yearly, monthly; weekly.

Only answer this question if the following conditions are met:
Answer was at question '23 [A18B]' (If you eat raw or lightly cooked fish: 18. B) Which fish? Please tick only the following fish in their corresponding box if you eat them.)

Please choose the appropriate response for each item:

	Yearly	Monthly	Weekly
Undercooked tuna (e.g. raw in middle)	○	○	○
Tuna with lemon or vinegar	○	○	○
Pickled or other marinating process	○	○	○
Cold-smocked	○	○	○
Sushi or sashimi	○	○	○
Ceviche	○	○	○
Dried	○	○	○
Brined or salted	○	○	○
Carpaccio	○	○	○
Other	○	○	○

You eat raw or lightly cooked **Haddock**:

18. C7) Which type of recipes or specialties? Multiple choices are possible.

Please tick only the following fish in their corresponding box if you eat them: yearly, monthly; weekly.

Only answer this question if the following conditions are met:
Answer was at question '23 [A18B]' (If you eat raw or lightly cooked fish: 18. B) Which fish? Please tick only the following fish in their corresponding box if you eat them.)

Please choose the appropriate response for each item:

	Yearly	Monthly	Weekly
Undercooked haddock (e.g. raw in middle)	○	○	○
Haddock with lemon or vinegar	○	○	○
Pickled or other marinating process	○	○	○
Cold-smoked haddock	○	○	○
Sushi or sashimi	○	○	○
Ceviche	○	○	○
Dried	○	○	○
Brined or salted	○	○	○
Carpaccio	○	○	○
Other	○	○	○

You eat raw or lightly cooked Cod:

18. C8) Which type of recipes or specialties? Multiple choices are possible.

Please tick only the following fish in their corresponding box if you eat them: yearly, monthly; weekly.

Only answer this question if the following conditions are met:
Answer was at question '23 [A18B]' (If you eat raw or lightly cooked fish: 18. B) Which fish? Please tick only the following fish in their corresponding box if you eat them.)

Please choose the appropriate response for each item:

	Yearly	Monthly	Weekly
Undercooked cod (e.g. raw in middle)	○	○	○
Cod with lemon or vinegar	○	○	○
Pickled or other marinating process	○	○	○
Cold-smoked cod	○	○	○
Sushi or sashimi	○	○	○
Ceviche	○	○	○
Dried	○	○	○
Brined or salted	○	○	○
Carpaccio	○	○	○
Other	○	○	○

You eat raw or lightly cooked Flatfish (e.g. plaice, sole, etc.):

18. C9) Which type of recipes or specialties? Multiple choices are possible.

Please tick only the following fish in their corresponding box if you eat them: yearly, monthly; weekly.

Only answer this question if the following conditions are met:
Answer was at question '23 [A18B]' (If you eat raw or lightly cooked fish: 18. B) Which fish? Please tick only the following fish in their corresponding box if you eat them.)

Please choose the appropriate response for each item:

	Yearly	Monthly	Weekly
Undercooked flatfish (e.g. raw in middle)	○	○	○
Flatfish with lemon or vinegar	○	○	○
Pickled or other marinating process	○	○	○
Cold-smoked	○	○	○
Sushi or sashimi	○	○	○
Ceviche	○	○	○
Dried	○	○	○
Brined or salted	○	○	○
Carpaccio	○	○	○
Other	○	○	○

You eat raw or lightly cooked Squid:

18. C10) Which type of recipes or specialties? Multiple choices are possible.

Please tick only the following fish in their corresponding box if you eat them: yearly, monthly; weekly.

Only answer this question if the following conditions are met:
Answer was at question '23 [A18B]' (If you eat raw or lightly cooked fish: 18. B) Which fish? Please tick only the following fish in their corresponding box if you eat them.)

Please choose the appropriate response for each item:

	Yearly	Monthly	Weekly
Undercooked squid (e.g. raw in middle)	○	○	○
Squid with lemon or vinegar	○	○	○
Pickled or other marinating process	○	○	○
Cold-smoked	○	○	○
Sushi or sashimi	○	○	○
Ceviche	○	○	○
Dried	○	○	○
Brined or salted	○	○	○
Carpaccio	○	○	○
Other	○	○	○

Only answer this question if the following conditions are met:
Answer was at question '23 [A18B]' (If you eat raw or lightly cooked fish: 18. B) Which fish? Please tick only the following fish in their corresponding box if you eat them.)

Please choose the appropriate response for each item:

○	○	○
○	○	○
○	○	○
○	○	○
○	○	○
○	○	○
○	○	○
○	○	○
○	○	○
○	○	○

You eat raw or lightly cooked **Other**:

18. C12) Please, write what kind of fish you consume and the frequency with which it does (yearly, monthly, weekly) in the appropriate box.

Only answer this question if the following conditions are met:
Answer was at question '23 [A18B]' (If you eat raw or lightly cooked fish: 18. B) Which fish? Please tick only the following fish in their corresponding box if you eat them.)

Please choose all that apply and provide a comment:

☐ Undercooked fish (e.g. raw in middle)	
☐ Fish with lemon or vinegar	
☐ Pickled or other marinating process (e.g. soused herring and rollmops)	
☐ Cold-smoked (e.g. salmon or trout)	
☐ Sushi or sashimi	
☐ Ceviche	
☐ Dried	
☐ Brined or salted (e.g. gravlax)	
☐ Carpaccio	
☐ Other	

Knowledge of Anisakis

19) Have you ever heard about *Anisakis* or “herring or cod worm” or “worms in fish”?

Please choose **only one** of the following:

- ○ Yes
- ○ No

20) Do you know about prevention methods against *Anisakis* or “herring or cod worm”?

Please choose **only one** of the following:

- ○ Yes
- ○ No

20. A) You answered yes to the previous question: Please pick the recommended prevention methods against worms in fish.

Only answer this question if the following conditions are met:
Answer was 'Yes' at question '37 [A20]' (20) Do you know about prevention methods against Anisakis or “herring or cod worm”?)

Please choose **all** that apply:

- ☐ Eat raw or lightly cooked fish
- ☐ Gutting the fish as soon as possible
- ☐ Cooking the fish
- ☐ Marinating or cold-smoking the fish
- ☐ Freezing the fish

21) Have you ever avoided buying or eating any fish due to the presence of *Anisakis*?

Please choose **only one** of the following:

- ○ Yes
- ○ No

21. A) You answered yes to the previous question, which fish have you avoided?

Only answer this question if the following conditions are met:
Answer was 'Yes' at question '39 [A21]' (21) Have you ever avoided buying or eating any fish due to the presence of Anisakis?)

Please choose **all** that apply:

- ☐ Hake
- ☐ Cod
- ☐ Herring
- ☐ Sardine
- ☐ Salmon or trout
- ☐ Whiting
- ☐ Tuna
- ☐ Blue whiting
- ☐ Flatfish (Plaice, sole, etc.)
- ☐ Mackerel
- ☐ Anchovy
- ☐ Other: ____________

Anisakis, anisakiasis and allergy to Anisakis

Please, **read carefully** the following information regarding *Anisakis* or “herring or cod worm” in fish products before answering the question.

***Anisakis* or “herring worm” is a natural worm in many marine fish.**

- **How to contract it: this worm may cause disease in humans who eat raw or lightly cooked infected fish products without prevention methods.**
- **Severity: This disease is called anisakiasis and its severity may vary from mild to severe. Other symptoms associated can be allergic reactions.**
- **Likelihood: This parasite is considered an emerging health problem worldwide, especially in Japan, Spain and Italy; where raw, lightly salted or marinated fish is part of the regular diet (sushi, sashimi, gravlax, salted or pickled herring, anchovies with vinegar, ceviche, etc.). Moreover, regarding food allergies, published data suggest that *Anisakis* is the major hidden producer of allergic reactions within Spain*. Only a few cases of human Anisakiasis were reported per year in Scotland, however no data about *Anisakis* allergy are available**
- **Prevention: To date, we know that freezing and/or cooking properly the fish kills the worm (*Anisakis*). However, it is common that people with allergy to *Anisakis* still show allergic symptoms after consuming fish that has been correctly frozen, cooked, and even processed.**

Note: Please visit EFSA (European Food Safety Authority) if you require more information about *Anisakis* and their related disease.

***Source: See the link. ***

Please choose **only one** of the following:

- Yes
- No

Please confirm that you have read this information

Willingness to pay

23) Please consider the following scenario and answer the question:

Suppose that a technology or treatment were discovered that guarantee that every *Anisakis* worms (and their related allergens) would be completely removed from fish, so the treatment was 100% effective. Suppose further that there are no side effects on fish quality.

Then, imagine that your favourite fish (e.g. cod) costs £6 per kilogram at the supermarket.

What is the maximum you would be willing and able to pay for the same fish product free of *Anisakis* fish worms and their allergens?

Please choose **only one** of the following:

- I would not buy this product
- 0% extra or £6 per kilogram (I will buy it at the stated price (£6))
- 10% extra or £6.60 per kilogram
- 20% extra or £7.20 per kilogram
- 30% extra or £7.80 per kilogram
- 40% extra or £8.40 per kilogram
- 50% extra or £9 per kilogram
- 60% extra or £9.60 per kilogram
- 70% extra or £10.20 per kilogram
- 80% extra or £10.80 per kilogram
- 90% extra or £11.40 per kilogram
- 100% extra or £12 per kilogram or more

23. A) If you are not willing to pay for this treatment, why?

Only answer this question if the following conditions are met:
Answer was 'I would not buy this product' *or* '0% extra or £6 per kilogram (I will buy it at the stated price (£6))' at question '42 [A23]' (23) Please consider the following scenario and answer the question: Suppose that a technology or treatment were discovered that guarantee that every Anisakis worms (and their related allergens) would be completely removed from fish, so the treatment was 100% effective. Suppose further that there are no side effects on fish quality. Then, imagine that your favourite fish (e.g. cod) costs £6 per kilogram at the supermarket. What is the maximum you would be willing and able to pay for the same fish product free of Anisakis fish worms and their allergens?)

Please choose **all** that apply:

- ☐ The risk is too small to warrant any price difference
- ☐ The scenario is not realistic
- ☐ You should not have to pay any premium for having safe food
- ☐ Other: __________

24) Would you avoid buying or eating a fish due to the presence of *Anisakis* or worms in fish?

Please choose **only one** of the following:

- ◯ Yes, always
- ◯ Yes, but only if there is high chance of worms
- ◯ No, cooking or freezing kill any possible worm

24. A) Why would you avoid the purchase or eating of these fish?

Only answer this question if the following conditions are met:
Answer was 'Yes, always' *or* 'Yes, but only if there is high chance of worms ' at question '44 [A24]' (24) Would you avoid buying or eating a fish due to the presence of Anisakis or worms in fish?)

Please choose **only one** of the following:

- ◯ Health risk
- ◯ Unappetising
- ◯ Both
- ◯ Other __________

Risk perception

25) Of course, no one can know for sure what will happen in the future, but we would like to know what you think about the following health risk:

Using a scale of 0-4 which 0 means no chance and 4 means absolutely certain,

what are the chances that you will develop an Anisakiasis and/or an allergy to *Anisakis*?

Please choose **only one** of the following:

- 0 (no chance)
- 1 (slight chance)
- 2 (moderate chance)
- 3 (high chance)
- 4 (absolutely chance)

Thank you very much for your kind cooperation!

Submit your survey.
Thank you for completing this survey.
